# Dietary silymarin improves performance by altering hepatic lipid metabolism and cecal microbiota function and its metabolites in late laying hens

**DOI:** 10.1186/s40104-024-01057-w

**Published:** 2024-07-13

**Authors:** Yanghao Guo, Yudong Xu, Derun Wang, Shihao Yang, Zehe Song, Rui Li, Xi He

**Affiliations:** 1 College of Animal Science and Technology, Hunan Agricultural University, Changsha, Hunan 410128 China; 2 Hunan Engineering Research Center of Poultry Production Safety, Changsha, Hunan 410128 China; 3 Yuelushan Laboratory, Hunan Agricultural University, Changsha, Hunan 410128 China; 4 Institute of Subtropical Agriculture, Chinese Academy of Sciences, Changsha, Hunan 410125 China

**Keywords:** Bile acid metabolism, Cecal microbiota, Laying hen, Lipid metabolism, Lipoproteins, Silymarin

## Abstract

**Background:**

Liver lipid dysregulation is one of the major factors in the decline of production performance in late-stage laying hens. Silymarin (SIL), a natural flavonolignan extracted from milk thistle, is known for its hepatoprotective and lipid-lowering properties in humans. This study evaluates whether SIL can provide similar benefits to late-stage laying hens. A total of 480 68-week-old Lohmann Pink laying hens were randomly assigned into 5 groups, each group consisting of 6 replicates with 16 hens each. The birds received a basal diet either without silymarin (control) or supplemented with silymarin at concentrations of 250, 500, 750, or 1,000 mg/kg (SIL250, SIL500, SIL750, SIL1000) over a 12-week period.

**Results:**

The CON group exhibited a significant decline in laying rates from weeks 9 to 12 compared to the initial 4 weeks (*P* = 0.042), while SIL supplementation maintained consistent laying rates throughout the study (*P* > 0.05). Notably, the SIL500 and SIL750 groups showed higher average egg weight than the CON group during weeks 5 to 8 (*P* = 0.049). The SIL750 group had a significantly higher average daily feed intake across the study period (*P* < 0.05), and the SIL500 group saw a marked decrease in the feed-to-egg ratio from weeks 5 to 8 (*P* = 0.003). Furthermore, the SIL500 group demonstrated significant reductions in serum ALT and AST levels (*P* < 0.05) and a significant decrease in serum triglycerides and total cholesterol at week 12 with increasing doses of SIL (*P* < 0.05). SIL also positively influenced liver enzyme expression (FASN, ACC, Apo-VLDL II, FXR, and CYP7A1; *P* < 0.05) and altered the cecal microbiota composition, enhancing species linked to secondary bile acid synthesis. Targeted metabolomics identified 9 metabolites predominantly involved in thiamin metabolism that were significantly different in the SIL groups (*P* < 0.05).

**Conclusions:**

Our study demonstrated that dietary SIL supplementation could ameliorate egg production rate in late stage laying hens, mechanistically, this effect was via improving hepatic lipid metabolism and cecal microbiota function to achieve. Revealed the potentially of SIL as a feed supplementation to regulate hepatic lipid metabolism dysregulation. Overall, dietary 500 mg/kg SIL had the best effects.

**Graphical Abstract:**

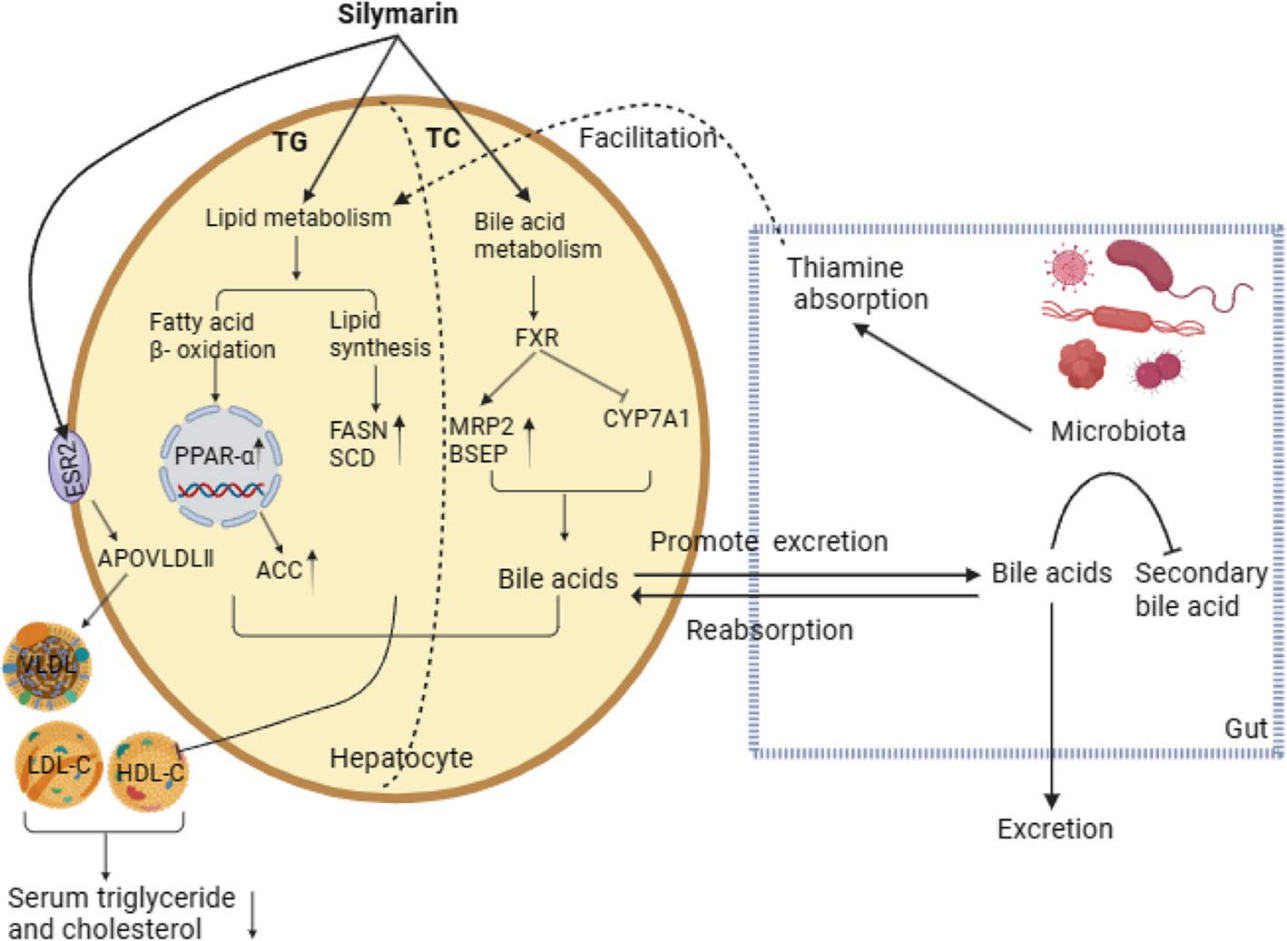

**Supplementary Information:**

The online version contains supplementary material available at 10.1186/s40104-024-01057-w.

## Introduction

Lipid metabolism dysfunction in late-stage laying hens is a significant contributor to the decline in both laying rates and egg quality, leading to substantial economic losses in the egg farming industry [1]. In poultry, the liver is the primary site of lipid metabolism, synthesizing over 90% of fatty acids. Very low-density lipoprotein (VLDL) plays a pivotal role in this process, transporting endogenous triglycerides (TG) from the liver to various tissues [2]. A specific subtype of VLDL, VLDLy, which includes apolipoprotein-VLDLII (ApoVLDLII) and apolipoprotein B100, is critical for moving endogenous triglycerides from the liver to the developing chicken oocyte [3]. Excessive fat accumulation in the liver can lead to lipid metabolic disorders and fatty liver hemorrhagic syndrome in hens [4, 5]. Thus, effectively regulating lipid metabolism in aging laying hens is essential for prolonging their productive lifespan and maintaining their health [6].

It was reported that estrogen levels may be a contributing factor to the disorders of lipid metabolism in late-stage laying hens [7]. Previous studies have demonstrated that serum estrogen levels in hens decrease with age after sexual maturity [8, 9]. Natural flavonoid, particularly isoflavone which exhibit xeno-estrogenic effects, capable of binding to estrogen receptors to elicit estrogenic or anti-estrogenic responses [10–14]. Moreover, dietary supplementation with genistein has been shown to alleviate fatty liver conditions in hyperlipidemic laying hens through up-regulation of estrogen receptor alpha (*ERα*) expression while inhibiting the expression of the NLR family pyrin domain containing 3 (*NLRP3*) inflammasome [11]. Additionally, the inclusion of quercetin in diets has been found to boost antioxidant status and hormone levels, thereby improving production performance in late-laying hens [13]. These findings suggest that dietary flavonoids may be an effective strategy for regulating lipid metabolism in aging laying hens.

Silymarin (SIL), a flavonolignan extracted from the seeds of milk thistle (*Silybum marianum L. Gaertn*), is renowned for its hepatoprotective properties, antioxidant activity, immunoregulatory effects, and its ability to regulate lipid metabolism [15–18]. It can bind to estrogen receptors across various tissues, leading to diverse physiological effects [19]. Our previous studies have shown that silymarin supplementation significantly influenced lipid and bile acid metabolism in yellow feather broilers [20, 21]. Faryadi et al. [22] reported that adding lecithinized silymarin and nano-silymarin could improve the production performance of laying hens, but this research didn't articulation the specific mechanisms. Additionally, recent findings indicate that silymarin mitigated obesity in mice fed a high-fat diet by modulating gut microbiota and its metabolites [23]. Nevertheless, the impact of SIL on the intestinal microbiota of late-stage laying hens has not been documented by any researchers. Building on these findings, we hypothesize that silymarin can improve production performance and egg quality in late-stage laying hens by regulating liver lipid metabolism and the metabolites of intestinal microbiota, and demonstrated the feasibility of the experiment and discussed the experimental dosage. This study aims to examine the dose-dependent effects of silymarin supplementation and to elucidate the underlying mechanisms by which silymarin alters lipid metabolism. These insights could provide a theoretical basis for enhancements in the laying hens farming industry.

## Materials and methods

### Birds and experimental design

Hunan Agricultural University Animal Ethics Committee (Changsha, China) reviewed and approved all experimental protocols (HAU ACC 2022176).

A total of 480 68-week-old Lohmann Pink layers with similar body weight (2.00 ± 0.12 kg) and laying rate (92.31% ± 1.12%) were randomly assigned to 5 groups (96 birds per group) with 6 replicates per group and 16 birds each replicate. The basal diet was not supplemented with other feed additives, coccidiostats, antibiotics, only non-starch polysaccharide enzyme, non-heat-treated powdered feed, and then the basal diet was additionally mixed with different levels of silymarin. The control (CON) group were fed with basal diet, and experimental groups were fed the basal diet with 250, 500, 750 or 1,000 mg/kg silymarin powder (SIL250, SIL500, SIL750, and SIL1000), respectively. The basal diet was formulated according to GB/T 5916–2020 recommendations (Table 1). All birds was raised in cages (188 cm × 34 cm × 37 cm) with 16 birds per cage in an environmentally controlled house that maintained at 24 ± 2 °C with a relative humidity of 45% to 60%. All the hens were free access to food and water with exposure to 16 h of light/d. Silymarin used in this study was provided by Inner Mongolia Ever Brilliance Biotechnology Co., Ltd. (Inner Mongolia, China). Notably, Silymarin is a standard extract from milk thistle, and its main components include silymarin as well as several flavonolignans, which are silymarin (47.09%), silybinA + B (27.82%), isosiybinA + B (5.24%), silydianin (12.01%), silychristin (1.93%), and the detailed information of the compounds were provided in Fig. S1.

**Table 1:** Ingredient composition and nutrient level of the basal diets (dry basis)

Ingredients	Content, %	Nutrient levels^2^	Content, %
Corn	40.07	ME, MJ/kg	10.80
Soybean meal	20.30	Crude protein	16.84
rough rice	20.00	Crude fat	2.96
Limestone	9.00	Crude fiber	2.43
Wheat bran	2.20	Calcium	3.95
Corn gluten meal (CGM)	2.00	Total phosphorus	0.66
Rapeseed meal	2.00	Available Calcium	3.56
Dicalcium phosphate (CaHPO_4_)	1.33	Available phosphorous	0.33
Soybean oil	0.60	Methionine	0.40
Premix^1^	2.50	Lysine	0.79
Total	100.00		

^1^The premix provided the following (per kilogram of diet): vitamin A, 10,000 IU; vitamin D_3_, 2,500 IU; vitamin E, 27.0 IU; vitamin K_3_, 3.0 mg; vitamin B_1_, 1.0 mg; vitamin B_2_, 4 mg; nicotinic acid, 32 mg; pantothenic acid, 11 mg; vitamin B_6_, 3.0 mg; folic acid, 0.5 mg; vitamin B_12_, 25 mg; biotin, 50.0 mg; Fe, 60.0 mg; Zn, 60.0 mg; Mn, 100.0 mg; Cu, 5.0 mg; I, 0.5 mg and Se, 0.2 mg

^2^The nutrient levels were calculated values

### Laying performance and egg quality analysis

At the whole trial period, the egg production and weight of each replicate were recorded daily, and feed intake were measured by replicate every 4 weeks (*n* = 96). Average daily feed intake (ADFI) and feed to egg ration were calculated every 4 weeks (*n* = 96). At the end of weeks 4, 8, and 12 of the trial, 5 eggs were randomly collected from each replicate (*n* = 30) for determining egg quality index including egg weight, albumen height, yolk color and haugh unit by ORKA EA-01 egg quality analyser (ORKA Food Technology Ltd., Herzeliya, IL) and determining eggshell strength by EFR-01 eggshell strength tester (ORKA Food Technology Ltd., Herzeliya, IL). Yolk weight, albumen weight and eggshell weight were measured by an electronic balance (SF-400, BAIJIE, China). Eggshell thickness without inner membrane was determined at blunt end, tip end and equatorial region using a vernier caliper (ARZ-1331, AIRAJ, China) and calculated the average of thickness. The eggshell brightness (L) value was measured once for each egg on the equatorial region by a precision colorimeter (NR20XE, 3nh, China).

### Serum, liver tissue and cecal content samples collection

At the end of the weeks 4, 8, and 12 of the trial, 1 bird from per replicate was randomly selected after 12 h food withdrawal to collect blood samples from the wing vein. Serum was separated from blood samples by centrifugation at 3,000 × *g* for 15 min using a centrifuge (TDZ4-WS, MKE, China) and then were stored at –20 °C for further analysis. The laying hens were killed by cervical dislocation and removed intact liver tissues to photograph on a clean background. After flushing the liver with saline, liver sample about 1 cm^3^ in size were collected from right lobe and placed in a 10% neutral formalin solution for liver morphology examination. A portion of the liver tissue was excised, immediately frozen in liquid nitrogen, and subsequently stored at –80 °C for mRNA analysis. The tip of the cecum was carefully excised from the laying hens, and the contents were expressed into sterile freezer tubes. These samples were then promptly frozen in liquid nitrogen and preserved at –80 °C, designated for subsequent metagenomic and targeted metabolomic analyses.

### Serum biochemical parameters

After the serum samples (*n* = 6) were pre-processed, serum total cholesterol (TC), triglycerides (TG), high-density lipoprotein-cholesterol (HDL-C), low-density lipoprotein-cholesterol (LDL-C), alanine aminotransferase (ALT) and aspartate aminotransferase (AST) were determined using the commercial assay kits (Nanjing Jiancheng Bioengineering Company, Jiangsu, China) according to the manufacturer’s protocols. Serum VLDL was analyzed using an ELISA kit (Shanghai Meilian Bio-technology Ltd., China) by enzyme-linked immunosorbent assay. And absorbance readings were taken for the treated samples using enzyme marker (Infinite M PLEX, Tecan, Switzerland).

### RNA isolation and real-time quantitative PCR

Total hepatic RNA was extracted from liver tissue (*n* = 6) using TRIzol reagent (Vazyme, Jiangsu, China) following the manufacturer’s instructions. The purity and concentration of RNA was determined using NanoDrop ND-1000 spectrophotometer (Thermo Fisher Scientific, Waltham, MA, USA). The high-capacity cDNA Reverse Transcription kit (AG, Hunan, China) was used for cDNA synthesis. Primer sequences used in the study were present in Table 2. Real-time quantitative PCR was conducted using SYBR Green Pro Taq HS (AG, Hunan, China) using a Real-time PCR machine (LightCycler 480 II, Roche, Switzerland). The reaction conditions were as follows: 50 °C for 2 min, 95 °C for 10 min; 40 cycles of 95 °C for 15 s, 60 °C for 1 min. Each sample was measured in duplicate and the relative mRNA expression of target genes was calculated using β-actin as an internal control by the 2^−ΔΔCT^ method. The Ct value of the target gene was obtained using RT-qPCR with *β-actin* as the internal gene. ΔCt = Ct (target gene) – Ct (*β-actin*). Normalized with the ΔCt of CON group, the variance multiplier was calculated by 2^−ΔΔCT^ in the final step.

**Table 2:** Primers used for RT-qPCR

Genes	Primer sequence(5´→3´)	GenBank ID	Product length, bp
*β-actin*	Forward: CCAGCCATGTATGTAGCCATCC Reverse: CACCATCACCAGAGTCCATCAC	NM_205518.2	88
*FXR*	Forward: TGGAGGCAACTGTGAGATGG Reverse: TGCCCATTTGCTTGCATTTCC	NM_24113.3	80
*CYP7A1*	Forward: GATCTTCCCAGCCCTTGTGG Reverse: AGCCTCTCCCAGCTTCTCAC	NM_001001753.2	82
*BSEP*	Forward: TGCAAAGCAAAGGAGACT Reverse: GCAATGGATAATGGAGGG	XM_040676679.2	193
*MRP2*	Forward: GGAGAGCAGTGATGCAAGTAGT Reverse: AGTTACTGAGCTGCCGATGC	NM_001012522.3	110
*ESR1*	Forward: TGGTACTACCGCTCCAGTGT Reverse: AGGCTGCTTGACCCAAAAGA	NM_205183.2	78
*ESR2*	Forward: TGCAGTGAACGACAAATTCAGA Reverse: TCCCTGTGAAGGCAAGACCT	NM_204794.3	82
*ACC*	Forward: ACATCCATCTTTGATGTGCT Reverse: AGGACATTCTGTTTGGGTG	XM_046929958.1	199
*FASN*	Forward: TGCTATGCTTGCCAACAGGA Reverse: ACTGTCCGTGACGAATTGCT	NM_205155.4	128
*SCD*	Forward: ACCTTAGGGCTCAATGCCAC Reverse: TCCCGTGGGTTGATGTTCTG	NM_204890.2	89
*SREBP-1*	Forward: TGGTGGTGGACGCCGAGAAG Reverse: GTCGTTGATGGATGAGCGGTAGC	NM_204126.3	134
*PPARα*	Forward: AGGCCAAGTTGAAAGCAGAA Reverse: TTTCCCTGCAAGGATGACTC	NM_001001464.1	155
*PPARγ*	Forward: TCTCCTGGCTTCTCTCAT Reverse: TGGGCTCCATAAAGTCAC	NM_001397666.1	116
*ELOVL6*	Forward: ACAAGGGCTTTTGGTGTCTCA Reverse: GGCCTACGGAGGCTTTTTGA	NM_001031539.2	101
*ELOVL7*	Forward: TTCACATGTGGTGCTCCATT Reverse: TGCTAAGGGCCATTTTCACCT	NM_001197310.1	177
*ApoB*	Forward: GGTTACTCCCACGATGGCAA Reverse: TCGCAGAAATGCCCTTCCTT	NM_001044633.2	120
*ApoVLDLII*	Forward: CTTAGCACCACTGTCCCTGAAGT Reverse: TGCATCAGGGATGACCAGC	NM_205483	81
*VTGII*	Forward: TTGCAAGCTGATGAACACACAC Reverse: GATTGCTTCATCTGCCAGGTC	NM_001031276	192
*GPR30*	Forward: AGGTCCAAGGATGTGCGCTGA Reverse: GTCGTAAGACCACGGCGGGA	NM_001162405.1	156

### Microbial DNA extraction and metagenomics analysis

Total microbial genomic DNA from cecal content (*n* = 6) was extracted using an OMEGA Soil DNA kit (D5625-01, OMEGA, USA). The quantity and quality of extracted DNA were measured using a NanoDrop ND-1000 spectrophotometer (Thermo Fisher Scientific, Waltham, MA, USA) and agarose gel electrophoresis, respectively. The extracted microbial DNA was processed to construct metagenome shotgun sequencing libraries with insert sizes of 400 bp by using Illumina TruSeq Nano DNA LT Library Preparation kit (Illumina, USA). Each library was sequenced by Illumina HiSeq X-ten platform (Illumina, USA) with PE150 strategy at Personal Biotechnology Co., Ltd. (Shanghai, China).

Raw sequencing reads were processed to obtain quality-filtered reads for further analysis. First, sequencing adapters were removed from sequencing reads using Cutadapt (v.1.2.1) [24]. Secondly, low quality reads were trimmed using a sliding-window algorithm in fastp [25]. Thirdly, reads were aligned to the host genome of broiler using BMTagger to remove host contamination. Once quality-filtered reads were obtained, taxonomical classifications of metagenomics sequencing reads from each sample were performed using Kraken2 [26] against an RefSeq-derived database, which included genomes from archaea, bacteria, viruses, fungi, protozoans, metazoans and Viridiplantae. CDS sequences of all samples were clustered by mmseqs2 [27] with “easy-cluster” mode, setting protein sequence identity threshold to 0.90 and covered residue of the shorter contig to 90%. The high-quality reads of each sample were aligned against the gene catalog by Salmon [28] to calculate relative gene abundance.

Beta diversity analysis was performed to investigate the compositional and functional variation of microbial communities across samples using Bray–Curtis distance metrics and visualized via principal coordinate analysis (PCoA). Based on the taxonomic and functional profiles of non-redundant genes, linear discriminant analysis effect size (LEfSe) was performed to detect differentially abundant taxa and functions across groups using the default parameters (*P* < 0.05, LDA thresholds > 2). The functionality of the non-redundant genes was obtained by annotated using MMseqs2 with the “search” mode against the protein databases of KEGG, EggNOG and CAZy, respectively, and using the Student's test method to compare the differences in abundance of each functional unit between sample groups. *P* < 0.05 was considered to be statistically significant, whereas a *P* < 0.10 was considered to constitute a tendency.

### Targeted metabolomics analysis of cecal chyme

The frozen cecal contents (*n* = 6) were mixed with pre-cooled methanol/acetonitrile/water solution (2:2:1, v/v). The mixture was milled (60 Hz, 2 min) and then subjected to ultrasonic extraction in an ice-water bath for 10 min. The mixture was allowed to stand at –20 °C for 10 min and then centrifuged for 20 min (14,000 ×* g*, 4 °C) and the supernatant was dried under vacuum. For mass spectrometry analysis, the sample was added with 100 μL of acetonitrile aqueous solution (acetonitrile:water = 1:1, v/v) and centrifuged for 15 min (14,000 × *g*, at 4 °C) and the supernatant was taken into the sample for analysis. Samples were separated on an Agilent 1290 Infinity LC UHPLC system with HILIC and C18 columns; the HILIC column temperature was 35 °C, the flow rate was 0.3 mL/min, and the injection volume was 2 μL; mobile phase composition A: 90% water + 2 mmol/L ammonium formate + 10% acetonitrile, B: acetonitrile + 0.4% formic acid. The AB 6500 + QTRAP mass spectrometer (AB SCIEX, USA) was used for mass spectrometry analysis. The ESI source conditions were as follows: source temperature: 580 °C, Ion Source Gas1 (GS1): 45, Ion Source Gas2 (GS2): 60, Curtain Gas (CUR): 35, IonSpray Voltage (IS): + 4,500 V or –4500 V in positive or negative modes, respectively, monitoring using the MRM model.

The peaks were extracted from the MRM raw data using MultiQuant or Analyst software. The ratio of the peak area of each substance to the peak area of the internal standard was then obtained, and the content was calculated according to the standard curve. Differential metabolites were identified by calculating the amount in the sample. The processed data were analyzed using the R software packages (ropls, V1.22.0; ggplot2, V3.4.1; pheatmap, V1.0.12; corrplot, V4.0.3), where they were subjected to multivariate data analysis, including principal component analysis (PCA) and orthogonal partial least squares discriminant analysis (OPLS-DA). Finally, the pathways in which differential metabolites were involved were obtained through metabolic pathway annotation in the KEGG database.

### Statistical analysis

Statistical analysis was performed with SPSS 26.0 software (IBM, USA). Data were tested for normal distribution using the Kolmogorov–Smirnov (K-S) test. The non-normally distributed data was transformed using their respective square roots, while the normally distributed data was left unchanged. Differences between groups were examined using one-way analysis of variance (ANOVA) with Duncan’s post hoc test, when the homogeneity of variance is not significant (*P* > 0.05). When the homogeneity of variance is significant (*P* < 0.05), using Welsh ANOVA test with Tamhane post hoc test. The linear and quadratic effects of dietary SIL supplementation dose were evaluated by regression analysis. All the results were expressed as mean ± standard deviation (SD). Differences were considered significant at *P* < 0.05.

## Results

### Production performance

**Fig. 1:**
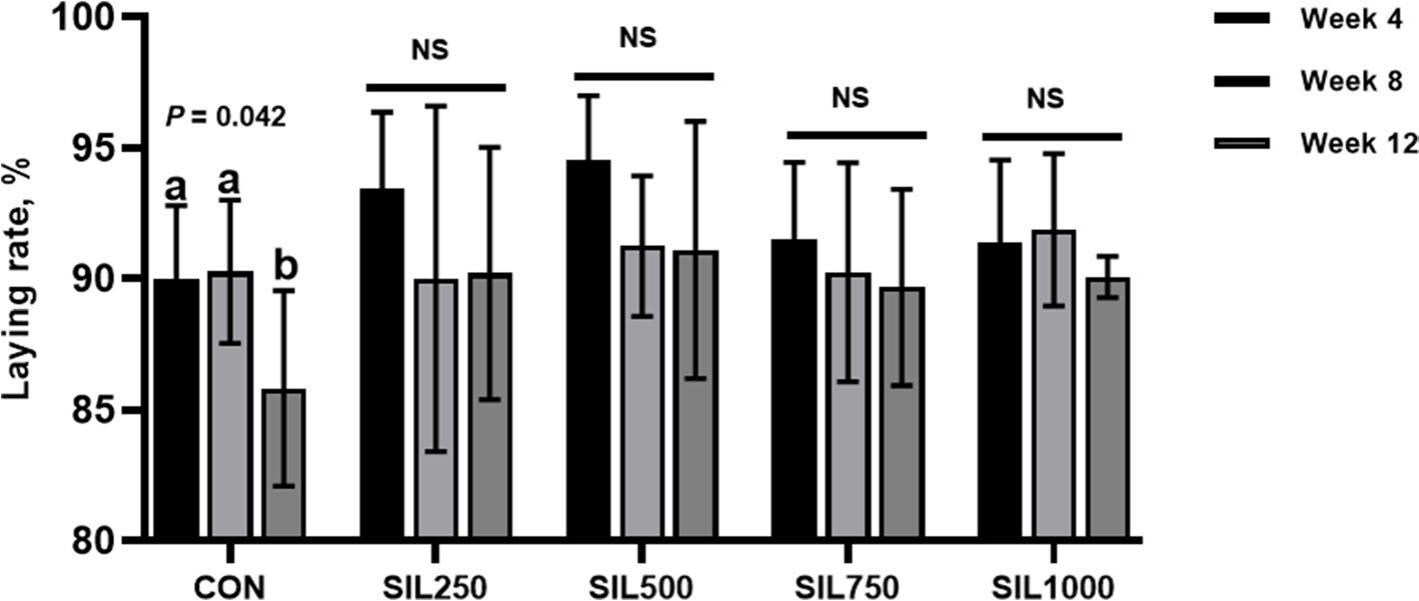
Changes in laying rate over the duration of the experiment in CON and SIL groups. ^a,b^Values with no common superscripts indicate a significant difference (*P* < 0.05), NS, no significant difference

Table 3 demonstrated that the dietary addition of SIL did not significantly influence the overall laying rate when compared to the CON group (*P* > 0.05). A quadratic increase in laying rate was observed (*P* = 0.017) with incremental additions of SIL during the first four weeks of the experiment trial. The laying rate in the CON group significantly decreased during weeks 9 to 12 compared to weeks 1 to 4 and weeks 5 to 8 (*P* = 0.042). In contrast, the groups receiving SIL supplementation exhibited no significant changes in laying rates between these time periods (*P* > 0.05), as shown in Fig. 1. Relative to the CON group, the treatments with SIL500 and SIL750 significantly enhanced average egg weight from weeks 5 to 8 (*P* = 0.049). Furthermore, the average daily feed intake for hens in the SIL750 group was significantly higher throughout the trial period compared to the CON group (*P* < 0.05). The feed-to-egg ratio in the SIL500 group showed a significant reduction from weeks 5 to 8 (*P* = 0.003).

**Table 3:** The effects in dietary silymarin inclusion on laying performance

Periods	Items	Groups					*P*-value		
		CON	SIL250	SIL500	SIL750	SIL1000	ANOVA	Linear	Quadratic
1–4 weeks	Laying rate, %	90.07 ± 2.78	93.44 ± 2.92	94.54 ± 2.45	91.51 ± 2.94	91.39 ± 3.15	0.084	0.849	0.017
	Average egg weight, g	63.92 ± 1.27	64.44 ± 1.32	64.71 ± 0.65	64.94 ± 1.47	63.79 ± 0.43	0.338	0.867	0.058
	ADFI, g/d/hen	120.57 ± 1.12^b^	121.54 ± 1.64^b^	121.51 ± 3.67^b^	126.74 ± 4.31^a^	123.75 ± 1.96^ab^	0.019	0.005	0.487
	Feed-egg ratio, g/g	1.94 ± 0.03^ab^	1.96 ± 0.08^ab^	1.89 ± 0.05^b^	2.00 ± 0.06^a^	1.98 ± 0.04^a^	0.022	0.116	0.316
5–8 weeks	Laying rate, %	90.27 ± 2.27	90.00 ± 6.59	91.25 ± 2.68	90.62 ± 4.18	91.87 ± 2.91	0.899	0.478	0.854
	Average egg weight, g	63.48 ± 0.99^b^	64.07 ± 0.88^ab^	64.90 ± 0.94^a^	64.98 ± 1.69^a^	63.45 ± 0.64^b^	0.049	0.546	0.006
	ADFI, g/d/hen	119.09 ± 4.00^b^	121.71 ± 2.77^ab^	116.90 ± 5.61^b^	125.14 ± 3.15^a^	119.46 ± 2.83^b^	0.034	0.407	0.548
	Feed-egg ratio, g/g	2.01 ± 0.05^a^	2.00 ± 0.07^a^	1.92 ± 0.06^b^	2.08 ± 0.04^a^	2.00 ± 0.07^a^	0.003	0.468	0.238
9–12 weeks	Laying rate, %	85.81 ± 3.73	90.20 ± 4.81	91.10 ± 4.91	89.68 ± 3.74	90.06 ± 0.79	0.231	0.125	0.094
	Average egg weight, g	64.66 ± 1.43	64.62 ± 0.68	65.33 ± 1.14	65.48 ± 1.45	63.68 ± 1.48	0.144	0.509	0.046
	ADFI, g/d/hen	112.00 ± 5.16^b^	117.57 ± 2.50^ab^	112.61 ± 6.96^b^	120.36 ± 4.86^a^	116.62 ± 4.72^ab^	0.040	0.070	0.461
	Feed-egg ratio, g/g	1.99 ± 0.13	2.07 ± 0.17	1.88 ± 0.08	2.05 ± 0.11	2.01 ± 0.09	0.105	0.834	0.571

*ADFI* Average daily feed intake, *SIL* Silymarin

^a,b^Within a row, values with no common superscripts indicate a significant difference (*P* < 0.05)

### Egg quality

As shown in Table 4, shell strength increased quadratically (*P* = 0.025) in response to the increasing addition of SIL by week 4. However, other indices did not differ significantly among the groups at week 4 (*P* > 0.05). By week 8, compared with CON group, the SIL500 and SIL750 treatments significantly improved eggshell thickness (*P* < 0.001). Meanwhile, the eggshell brightness in the SIL750 and SIL1000 groups was lower (*P* = 0.013). By week 12, the SIL1000 treatment had significantly reduced the eggshell thickness, eggshell weight (*P* < 0.001), and eggshell brightness (*P* = 0.003) compared with the CON group.

**Table 4:** The effect of silymarin on the egg quality of laying hens

Periods	Items	Group					*P*-value		
		CON	SIL250	SIL500	SIL750	SIL1000	ANOVA	Linear	Quadratic
week 4	Shell strength, kg/cm^2^	4.03 ± 0.21^c^	4.60 ± 0.30^a^	4.25 ± 0.38^ab^	4.46 ± 0.34^ab^	4.18 ± 0.28^bc^	0.025	0.680	0.021
	Egg weight, g	64.36 ± 2.24	64.33 ± 1.66	65.12 ± 0.71	64.94 ± 0.72	64.05 ± 1.04	0.651	0.999	0.222
	Albumen height, mm	5.29 ± 0.25	5.28 ± 0.24	5.22 ± 0.44	6.03 ± 0.69	5.29 ± 1.17	0.264	0.379	0.575
	Yolk, color	8.72 ± 2.17	9.80 ± 0.52	10.40 ± 1.12	10.44 ± 1.17	9.6 ± 0.61	0.390	0.238	0.113
	Haugh unit	68.60 ± 5.14	70.19 ± 4.84	67.48 ± 4.43	74.44 ± 5.72	67.29 ± 12.96	0.447	0.865	0.493
	Albumen weight, g	33.29 ± 2.36	34.74 ± 0.91	34.84 ± 0.54	35.45 ± 0.79	34.97 ± 1.26	0.097	0.027	0.115
	Yolk weight, g	18.80 ± 0.91	18.87 ± 0.76	19.03 ± 0.66	18.92 ± 0.76	18.53 ± 0.41	0.806	0.608	0.295
	Eggshell thickness, μm	364.79 ± 9.47	343.00 ± 17.73	347.93 ± 19.23	355.78 ± 12.14	352.00 ± 4.26	0.100	0.476	0.075
	Eggshell weight, g	6.47 ± 0.41	6.57 ± 0.21	6.54 ± 0.22	6.54 ± 0.17	6.27 ± 0.12	0.238	0.194	0.067
	Eggshell brightness L	84.75 ± 0.62	85.76 ± 1.80	84.95 ± 0.90	84.14 ± 1.66	84.02 ± 0.13	0.509	0.110	0.773
week 8	Shell strength, kg/cm^2^	4.30 ± 0.14	4.36 ± 0.33	4.27 ± 0.33	4.16 ± 0.60	4.22 ± 0.30	0.912	0.481	0.972
	Egg weight, g	64.97 ± 3.21	65.25 ± 1.63	66.23 ± 1.31	66.21 ± 1.37	63.97 ± 1.29	0.090	0.676	0.049
	Albumen height, mm	5.42 ± 0.56	5.30 ± 0.37	5.56 ± 0.59	5.17 ± 0.44	5.57 ± 0.34	0.534	0.765	0.592
	Yolk, color	12.30 ± 0.55	12.73 ± 0.70	12.87 ± 0.74	12.63 ± 0.43	12.63 ± 0.46	0.564	0.464	0.184
	Haugh unit	69.22 ± 5.51	68.41 ± 4.13	70.31 ± 4.82	66.49 ± 4.54	71.57 ± 2.93	0.371	0.634	0.382
	Albumen weight, g	34.19 ± 2.34	34.97 ± 1.15	33.66 ± 4.05	34.96 ± 1.33	34.15 ± 1.26	0.833	0.974	0.867
	Yolk weight, g	18.76 ± 1.04	18.89 ± 0.65	18.96 ± 0.91	19.30 ± 0.79	18.60 ± 0.49	0.637	0.946	0.268
	Eggshell thickness, μm	309.17 ± 19.61^b^	258.67 ± 16.32^c^	363.35 ± 18.96^a^	350.89 ± 19.72^a^	289.33 ± 24.55^b^	< 0.001	0.052	< 0.001
	Eggshell weight, g	6.62 ± 0.36	6.69 ± 0.19	6.99 ± 0.29	6.86 ± 0.20	6.78 ± 0.28	0.180	0.187	0.089
	Eggshell brightness L	85.05 ± 1.29^a^	83.39 ± 1.08^ab^	84.65 ± 1.58^a^	82.40 ± 2.08^b^	82.84 ± 0.61^b^	0.013	0.007	0.750
week 12	Shell strength, kg/cm^2^	4.20 ± 0.41	4.15 ± 0.32	3.56 ± 0.72	3.88 ± 0.21	3.94 ± 0.39	0.134	0.182	0.112
	Egg weight, g	63.38 ± 1.88	65.37 ± 2.21	64.21 ± 1.71	65.15 ± 1.32	62.82 ± 2.48	0.145	0.603	0.039
	Albumen height, mm	6.47 ± 0.42	6.22 ± 0.66	6.31 ± 0.39	6.61 ± 0.98	6.06 ± 0.45	0.581	0.602	0.667
	Yolk, color	13.23 ± 0.45	13.35 ± 0.22	13.20 ± 0.55	13.02 ± 0.61	12.96 ± 0.33	0.561	0.143	0.584
	Haugh unit	78.78 ± 2.82	76.18 ± 4.65	77.42 ± 2.54	78.91 ± 6.61	75.97 ± 3.07	0.633	0.599	0.947
	Albumen weight, g	34.34 ± 2.25	35.61 ± 2.34	34.93 ± 1.23	36.36 ± 1.27	34.86 ± 1.71	0.379	0.453	0.230
	Yolk weight, g	19.01 ± 0.50^ab^	19.64 ± 0.64^a^	19.04 ± 0.79^ab^	18.74 ± 0.49^b^	18.36 ± 0.73^b^	0.030	0.014	0.088
	Eggshell thickness, μm	349.44 ± 10.89^ab^	346.22 ± 8.54^ab^	357.60 ± 15.20^a^	336.89 ± 8.52^ab^	319.89 ± 10.01^b^	< 0.001	< 0.001	< 0.001
	Eggshell weight, g	7.04 ± 0.18^a^	7.13 ± 0.13^a^	7.24 ± 0.38^a^	7.05 ± 0.40^a^	6.61 ± 0.25^b^	0.011	0.018	0.005
	Eggshell brightness L	84.39 ± 1.20^a^	85.02 ± 1.76^a^	86.33 ± 2.03^a^	84.67 ± 1.69^a^	81.89 ± 1.86^b^	0.003	0.024	0.001

^a–c^Within a row, values with no common superscripts indicate a significant difference (*P* < 0.05)

### Serum biochemistry

As shown in Table 5, at week 4, serum AST activity was significantly lower in the SIL250 and SIL750 groups compared to the CON group (*P* = 0.002), with a quadratic decline observed in AST activity (*P* = 0.005). Serum VLDL content increased linearly (*P* < 0.001) in response to increasing doses of SIL. By week 8, serum AST activity in the SIL500 and SIL750 groups was significantly improved compared to other groups (*P* < 0.001), with AST activity increasing quadratically (*P* = 0.007). By week 12, the SIL500 group exhibited a decrease in serum AST activity compared to other groups (*P* = 0.002). Additionally, with increasing doses of SIL, there was a linear decrease in both serum ALT activity (*P* = 0.013) and VLDL content (*P* < 0.001). Serum ALT activity in the SIL500 and SIL1000 groups was significantly lower than in the CON group at week 12 (*P* = 0.048).

**Table 5:** The effect of silymarin on the serum biochemistry of laying hens

Periods	Items	Group					*P*-value		
		CON	SIL250	SIL500	SIL750	SIL1000	ANOVA	Linear	Quadratic
week 4	AST, U/L	42.43 ± 13.94^a^	21.50 ± 9.54^b^	30.60 ± 10.45^ab^	26.42 ± 6.74^b^	31.97 ± 8.70^ab^	0.002	0.322	0.005
	ALT, U/L	3.17 ± 0.33	2.90 ± 0.24	3.32 ± 1.00	3.30 ± 0.45	3.16 ± 0.57	0.309	0.615	0.819
	TC, mmol/L	4.35 ± 1.25	5.28 ± 0.72	5.42 ± 1.89	5.45 ± 1.31	6.02 ± 1.67	0.390	0.069	0.706
	TG, mmol/L	4.99 ± 1.12^b^	5.98 ± 2.05^b^	5.76 ± 1.33^b^	5.86 ± 1.00^b^	7.75 ± 1.35^a^	0.034	0.007	0.339
	HDL-C, mmol/L	3.37 ± 1.06	3.03 ± 0.47	2.59 ± 0.58	2.96 ± 0.32	2.96 ± 0.79	0.452	0.331	0.171
	LDL-C, mmol/L	1.65 ± 0.33^b^	2.58 ± 0.47^a^	1.69 ± 0.39^b^	2.32 ± 0.43^a^	2.56 ± 0.43^a^	< 0.001	< 0.001	0.803
	VLDL, mmol/L	19.84 ± 1.78^b^	20.09 ± 2.00^b^	23.22 ± 0.76^a^	22.93 ± 1.43^a^	24.11 ± 2.06^a^	< 0.001	< 0.001	0.546
week 8	AST, U/L	23.56 ± 2.31^b^	24.43 ± 5.88^b^	44.54 ± 12.58^a^	48.98 ± 9.99^a^	28.21 ± 7.55^b^	< 0.001	< 0.001	< 0.001
	ALT, U/L	2.97 ± 0.97	3.30 ± 1.18	2.90 ± 0.77	3.09 ± 0.33	3.07 ± 0.77	0.862	0.319	0.969
	TC, mmol/L	4.40 ± 1.58	5.08 ± 1.65	5.23 ± 1.76	5.77 ± 1.37	4.10 ± 1.51	0.392	0.974	0.087
	TG, mmol/L	5.31 ± 1.72	5.91 ± 1.23	6.03 ± 1.40	7.43 ± 2.34	5.41 ± 1.43	0.219	0.429	0.133
	HDL-C, mmol/L	4.30 ± 1.13	3.53 ± 1.15	3.31 ± 0.87	4.14 ± 1.54	3.56 ± 1.15	0.549	0.572	0.439
	LDL-C, mmol/L	2.37 ± 0.55	2.24 ± 0.46	2.21 ± 0.42	2.25 ± 0.58	1.97 ± 0.52	0.745	0.240	0.772
	VLDL, mmol/L	24.44 ± 5.05	23.10 ± 2.19	25.11 ± 2.80	25.45 ± 3.59	25.18 ± 3.02	0.760	0.395	0.920
week 12	AST, U/L	23.81 ± 4.77^a^	30.17 ± 7.03^a^	16.10 ± 2.40^b^	23.96 ± 4.99^a^	25.05 ± 5.18^a^	0.002	0.578	0.154
	ALT, U/L	3.96 ± 0.51^a^	3.71 ± 0.32^ab^	2.98 ± 0.54^b^	3.36 ± 0.78^ab^	3.10 ± 0.75^b^	0.048	0.013	0.246
	TC, mmol/L	8.94 ± 1.58^a^	6.51 ± 2.09^ab^	4.77 ± 1.82^b^	5.66 ± 1.96^b^	5.89 ± 1.78^b^	0.023	0.018	0.019
	TG, mmol/L	8.61 ± 1.32^a^	7.00 ± 0.86^b^	6.19 ± 1.03^b^	6.45 ± 1.46^b^	7.60 ± 1.33^ab^	0.015	0.114	0.002
	HDL-C, mmol/L	8.53 ± 2.03^a^	6.76 ± 1.74^b^	6.84 ± 0.92^b^	5.42 ± 0.98^bc^	4.87 ± 0.73^c^	0.005	< 0.001	0.658
	LDL-C, mmol/L	2.30 ± 0.47^a^	1.86 ± 0.51^a^	1.32 ± 0.21^b^	1.26 ± 0.28^bc^	0.84 ± 0.31^c^	0.001	< 0.001	0.368
	VLDL, mmol/L	17.87 ± 0.98^b^	15.55 ± 2.48^b^	22.61 ± 2.9^a^	15.73 ± 1.64^b^	22.94 ± 1.05^a^	< 0.001	< 0.001	0.100

*ALT* Alanine aminotransferase, *AST* Aspartate aminotransferase, *TC* Total cholesterol, *TG* Triglycerides, *HDL-C* High-density lipoprotein cholesterol, *LDL-C* Low-density lipoprotein cholesterol, *VLDL* Very-low-density lipoprotein, *E2* Estradiol

^a–c^Within a row, values with no common superscripts indicate a significant difference (*P* < 0.05)

Regarding serum lipid profiles, at week 4, serum TG levels in the SIL1000 group were significantly higher than in the other groups (*P* = 0.034) and LDL-C content increased linearly with increasing doses of SIL (*P* < 0.001). At week 12, TC and TG levels decreased quadratically (*P* < 0.05), while HDL-C and LDL-C content decreased linearly (*P* < 0.001) with increasing additions of SIL. At week 12, the dietary inclusion of 500, 750, or 1,000 mg/kg of silymarin significantly reduced serum TC (*P* = 0.023) and LDL-C content (*P* < 0.001) compared to the CON group. Serum TG levels in the SIL250, SIL500, and SIL750 groups were significantly lower than in the CON group (*P* = 0.015). Compared to the CON group, the addition of silymarin in the diet led to a decrease in serum HDL-C concentration (*P* < 0.001) and an increase in serum VLDL content (*P* < 0.001).

### Liver lipid metabolism

**Fig. 2:**
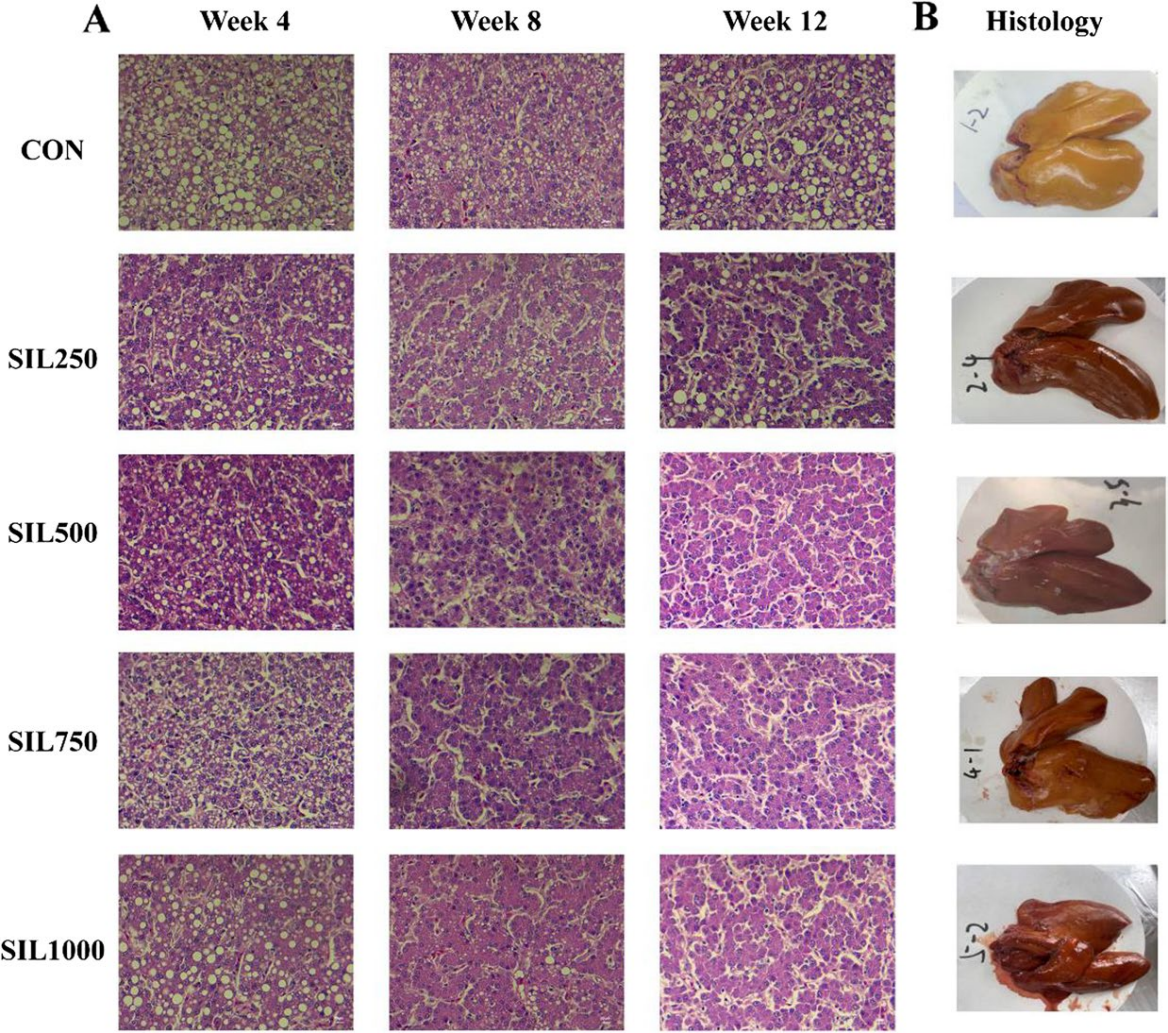
The effect of dietary silymarin on hepatic histomorphology of laying hens. **A** The effect of dietary silymarin on hepatic histomorphology H&E stained sections of laying hens (400 × magnification). **B** Liver images at week 12

As illustrated in Fig. 2, H&E staining of liver sections from the CON group revealed lipid vacuolization and severe steatosis in hepatocytes (Fig. 2A). Silymarin treatment effectively ameliorated liver steatosis and reduced lipid vacuolization within hepatocytes.

**Fig. 3:**
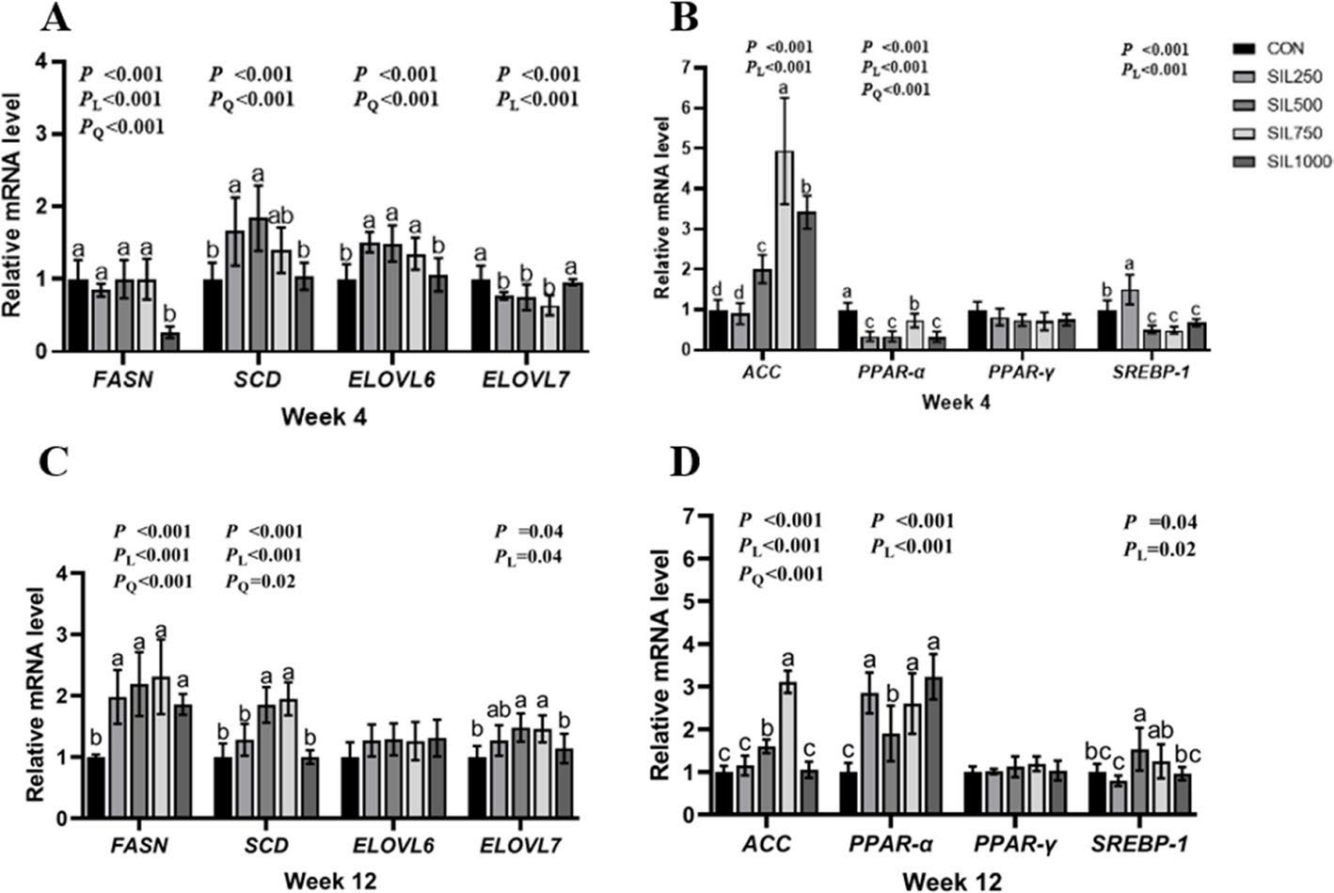
The effect of silymarin on liver lipid metabolism of late laying hens. **A** and **B** The effect of silymarin on liver lipid metabolism of laying hens at the fourth week. **C** and **D** The effect of silymarin on liver lipid metabolism of laying hens at the twelfth week. ^a–c^values with no common superscripts indicate a significant difference (*P* < 0.05). *FASN* Fatty acid synthase, *SCD* Stearoyl-CoA desaturase, *ELOVL6* Elongation of very long chain fatty acids 6, *ELOVL7* Elongation of very long chain fatty acids 7, *ACC* Acetyl-CoA carboxylase, *PPAR-α* Peroxisome proliferators-activated receptor-α, *PPAR-γ* Peroxisome proliferators-activated receptor-γ, *SREBP-1* Sterol-regulatory element binding protein-1

The impact of silymarin on the expression of lipid synthesis-related genes is depicted in Fig. 3A and C. The expression of fatty acid synthase *(FASN*) was regulated quadratically throughout all trial periods (*P* < 0.001), with significant downregulation observed in the SIL1000 group at week 4 (*P* < 0.001). By week 12, *FASN* expression in all SIL groups was significantly upregulated compared to the CON group (*P* < 0.001). The mRNA expression of stearoyl-CoA desaturase (*SCD*) also increased quadratically during all trial periods (*P* < 0.001), with significant increases in the SIL250 and SIL500 groups at week 4 and in the SIL500 and SIL750 groups at week 12 compared to the CON group (*P* < 0.001). Supplementation with 250, 500, or 750 mg/kg silymarin significantly improved the expression of elongation of very long-chain fatty acids protein 6 (*ELOVL6*) and inhibited *ELOVL7* expression at week 4. However, *ELOVL7* expression in the SIL500 and SIL750 groups was significantly lower than in the CON group at week 12 (*P* < 0.001).

As shown in Fig. 3B and D, mRNA expression of acetyl-CoA carboxylase (*ACC*) increased linearly in all trial periods (*P* < 0.001), with the SIL500 and SIL750 groups exhibiting significantly higher levels than the CON group at both weeks 4 and 12 (*P* < 0.001). Dietary silymarin addition significantly decreased the expression of peroxisome proliferator-activated receptor-α (PPAR-α) at week 4, but increased its expression by week 12. The expression of sterol-regulatory element-binding protein-1 (SREBP-1) was linearly regulated throughout all trial periods (*P* < 0.001), with the SIL250 group showing significantly higher expression, whereas the SIL500, SIL750, and SIL1000 groups had lower expression than the CON group at week 4 (*P* < 0.001). Moreover, *SREBP-1* expression in the SIL500 group was significantly higher than in the CON, SIL250, and SIL1000 groups at week 12 (*P* = 0.04).

### Lipoprotein expression

**Fig. 4:**
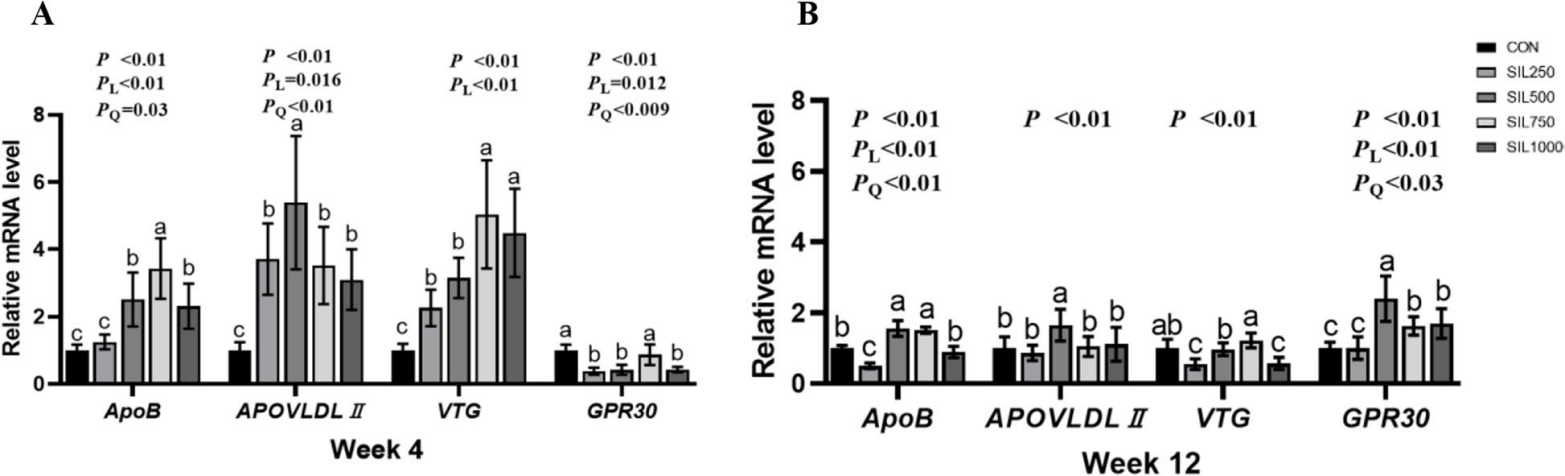
The effect of dietary silymarin on liver lipoprotein synthesis of laying hens. **A** The effect of silymarin on liver Lipoprotein synthesis of laying hens at the fourth week. **B** The effect of silymarin on liver Lipoprotein synthesis of laying hens at the twelfth week. ^a–c^Values with no common superscripts indicate a significant difference (*P* < 0.05). *ApoB* Apolipoprotein B, *ApoVLDLII* Apolipoprotein Very Low Density LipoproteinII, *VTG* Vitellogenesis, *GPR30* G protein-coupled estrogen receptor 30

At weeks 4 and 12, the apolipoprotein B (*ApoB*) expression regulated quadratically (*P* < 0.05) and SIL500, SIL750 groups were significantly higher than CON group and SIL250 group (*P* < 0.001) in all trial periods. *ApoVLDLII* and vitellogenesis (*VTG*) expression in all SIL groups were significantly higher than CON group at week 4 (*P* < 0.01). Compared with CON group, the expression of G protein-coupled estrogen receptor 30 (*GPR30*) regulated linearly in all trial periods (*P* < 0.001) and SIL250, SIL500 and SIL1000 groups were significantly reduced at week 4 (*P* < 0.001). *ApoVLDLII *and *GPR30* expression in SIL500 group were significantly higher than other groups at week 12 (*P* < 0.01). *VTG* expression in SIL750 group were significantly higher than other groups at week 12 (*P* < 0.001) (Fig. 4A and B).

### Liver bile acids metabolism and estrogen receptors expression

**Fig. 5:**
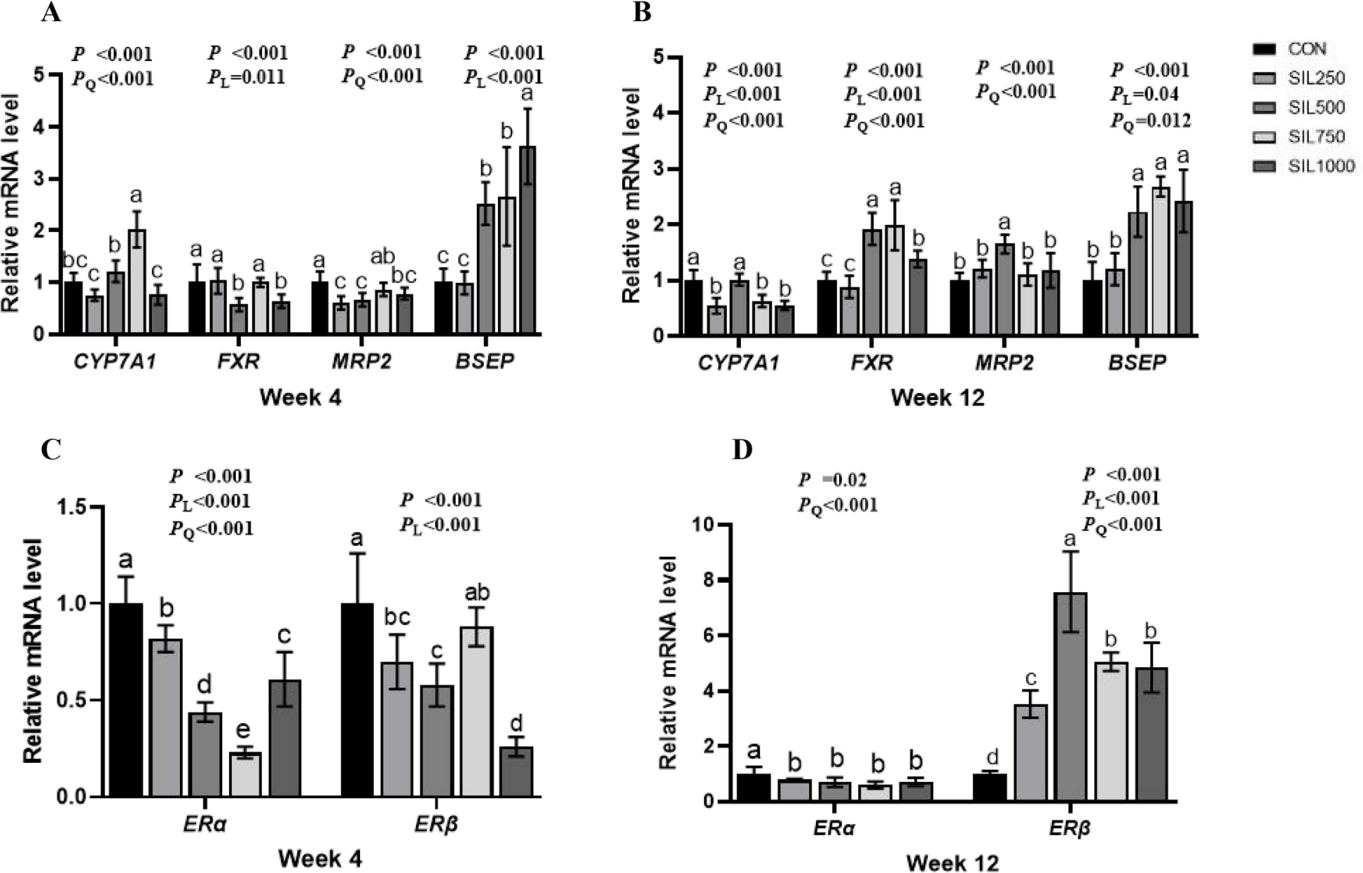
The effect of dietary silymarin on liver BAs metabolism and ER expression in laying hens. **A** The effect of silymarin on liver bile acid metabolism of laying hens at the fourth week. **B** The effect of silymarin on liver bile acid metabolism of laying hens at the twelfth week. **C** The effect of dietary silymarin on liver *ERα* expression in laying hens. **D** The effect of dietary silymarin on liver *ERβ* expression in laying hens. ^a–d^Values with no common superscripts indicate a significant difference (*P* < 0.05). *ERα* Estrogen receptor α, *ERβ* Estrogen receptor β, *CYP7A1* Cholesterol 7alpha-monooxygenase, *FXR* Farnesoid X receptor, *MRP2* Multidrug resistance associated-protein, *BSEP* Bile salt export pump

The mRNA expression of cholesterol 7 alpha-monooxygenase (*CYP7A1*) increased quadratically in all trial periods (*P* < 0.001) and in SIL250 and SIL1000 groups were significantly lower than other groups at weeks 4 and 12 (*P* < 0.001). The farnesoid X receptor (*FXR*) expression in SIL500 and SIL1000 were lower than other groups at week 4 (*P* < 0.001). The expression of *FXR* levels increased quadratically (*P* < 0.001), SIL500, SIL750 and SIL1000 groups were significantly higher than CON and SIL250 groups at week 12 (*P* < 0.001). The multidrug resistance associated-protein (*MRP2*) expression in SIL250, SIL500 and SIL1000 were significantly lower than the CON group at week 4 (*P* < 0.001), but at week 12 its expression in SIL750 were significantly higher than other groups (*P* < 0.001). Compared with CON group and SIL250 group, the bile salt export pump (*BSEP*) expression increased linearly in all trial periods (*P* < 0.001) and SIL500, SIL750 and SIL1000 groups were significantly increased (*P* < 0.001) at weeks 4 and 12 (Fig. 5A and B).

Compared with the CON group, SIL treatment significantly reduced estrogen receptor α (*ERα*) expression at the weeks 4 and 12 (*P* < 0.001) (Fig. 5C and D). The mRNA expression of estrogen receptor β (*ERβ*) in SIL250, SIL500 and SIL100 groups were significantly downregulated compared to CON group at week 4 (*P* < 0.001). At week 12, the expression of *ERβ* in all SIL groups were significantly improved relative with CON group (Fig. 5C and D) (*P* < 0.001).

### Microbial composition and function

**Fig. 6:**
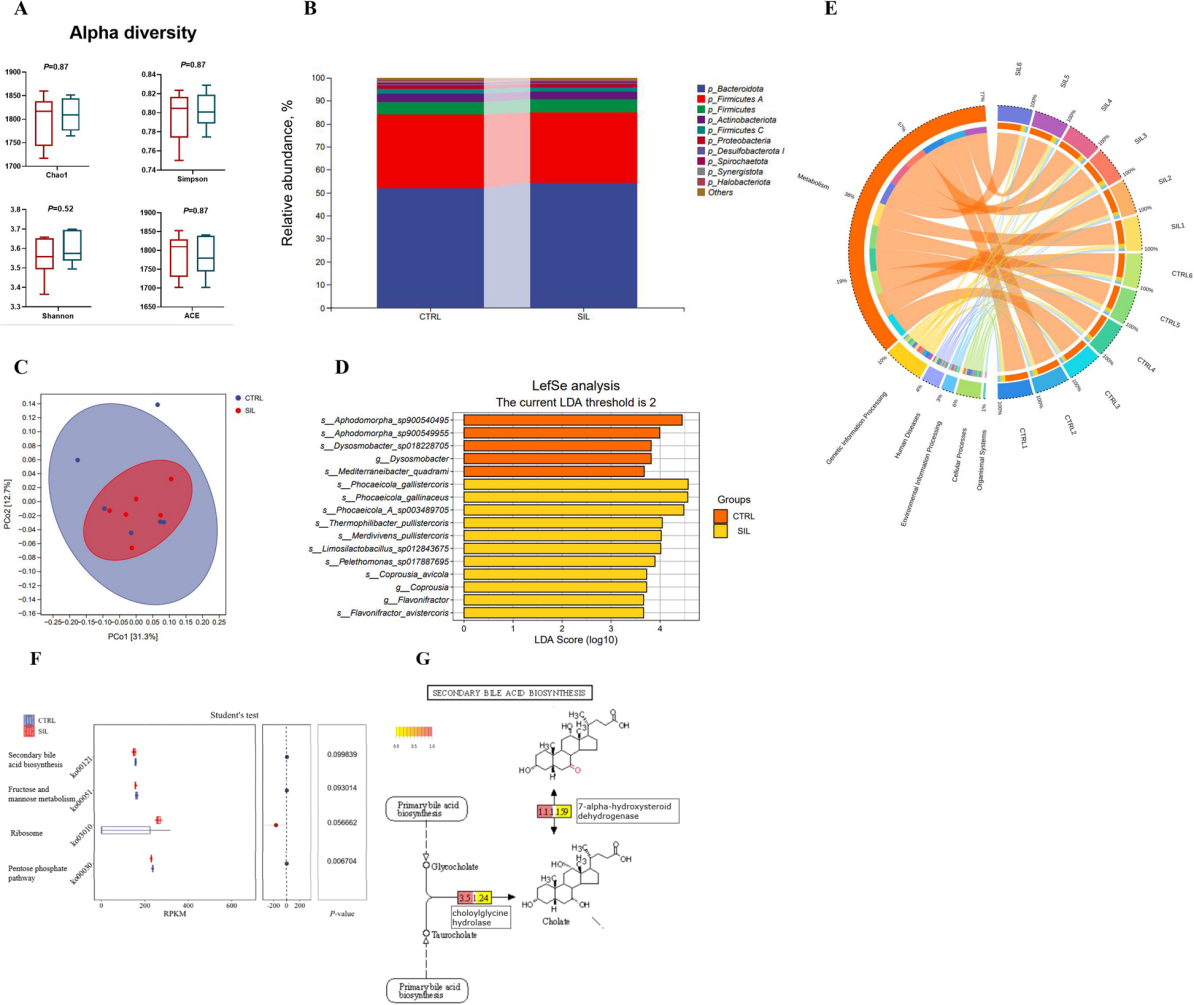
Diversity analysis, differential and functional enrichment analysis of cecal microbes. **A** The alpha diversity of cecal microbiota was analyzed by Chao1, Simpson, Shannon, ACE. Red is CON group and blue is the SIL group. **B** Differences of cecal flora at phylum level. **C** The beta diversity of cecal microbiota was analyzed by PCoA. **D** The different microbial groups in cecum of laying hens were analyzed based on LEfSe method. **E** The circos map of functional component relationships **F** CON group vs. SIL group comparison between different functional units. **G** The comparison of gene expression enriched in secondary bile acid biosynthesis (pink for CON group, yellow for SIL group). PCoA, Principal coordinates analysis; LEfSe, Linear discriminant analysis effect size

As shown in Fig. 6A, alpha diversity analysis including Chao1, ACE, Shannon and Simpson were not significant between CON and SIL groups (*P* > 0.05). PCoA showed that there was no obvious separation between CON and SIL groups (Fig. 6C). Bacteroidota, Firmicutes and Actinobacteria were ranked as the top 3 bacteria at the phylum level(Fig. 6B). LefSe analysis showed that *Phocaeicola_gallinaceus*, *Phocaeicola_pullistercoris*, *Limosilactobacillus_sp012843675, Pelethomonas_sp017887695, Coprousia_avicola* and *Flavonifractor_avistercoris* were regarded as the dominant bacteria in SIL group (Fig. 6D). Functional difference analysis showed four pathways that tended to be significant different between CON group and SIL group (*P* < 0.10), including secondary bile acid biosynthesis (ko00121), fructose and mannose metabolism (ko00051), ribosome (ko00030) and Pentose phosphate pathway (ko00030) (Fig. 6F). We further analyzed the expression of microbial genes involved in secondary bile acid metabolism pathway and found that choloylglycine hydrolase and 7-alpha-hydroxysteroid dehydrogenase were down-regulated (Fig. 6G).

### Metabolomic profiling of the cecum

**Fig. 7:**
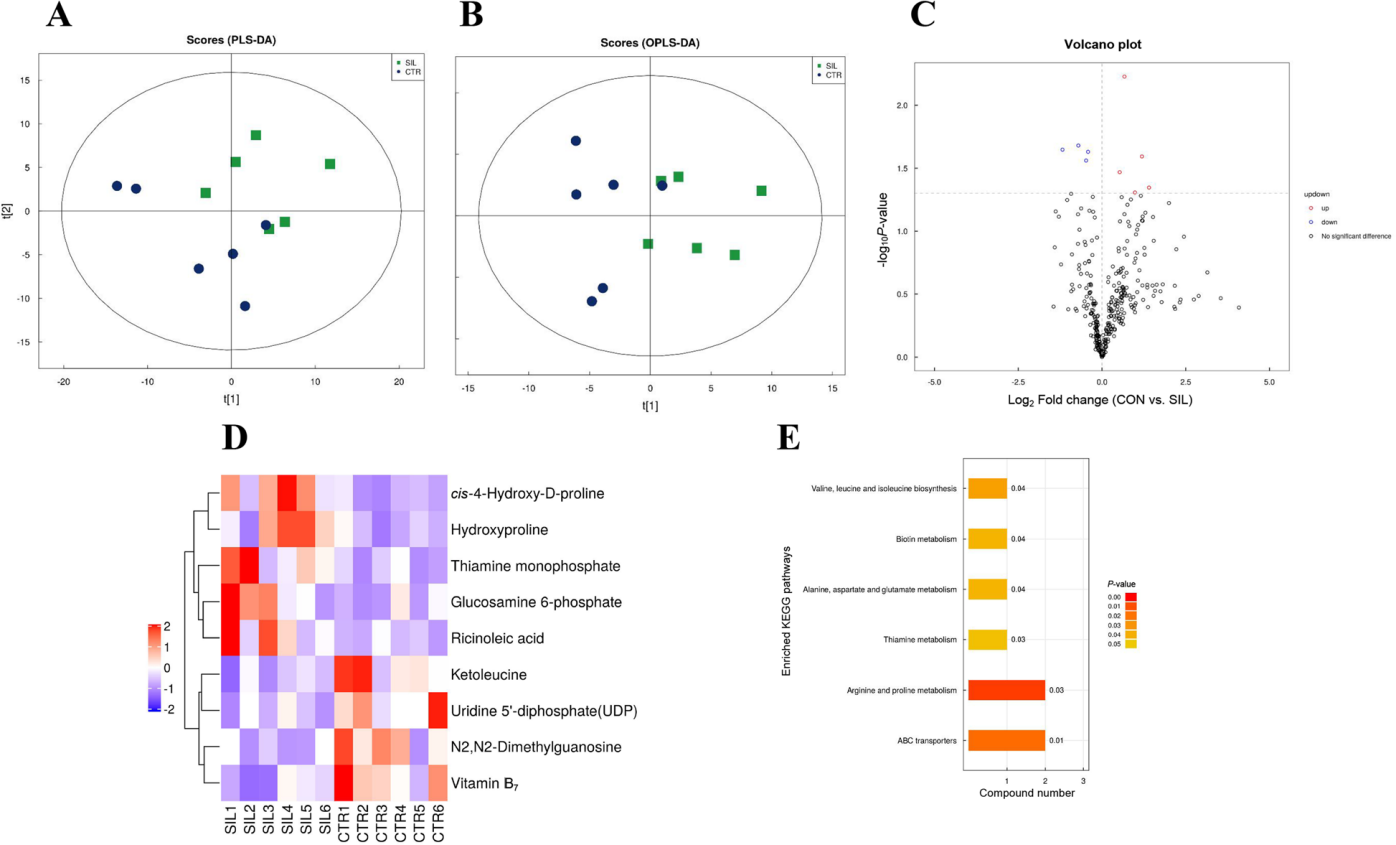
Metabolic alterations induced by silymarin. **A** PLS-DA between the SIL and CON groups. **B** OPLS-DA score plot between the SIL and CON groups. **C** Volcano plot shows the different metabolites between the SIL and CON groups. **D** Heat map shows the alteration patterns of significantly changed metabolites between the SIL and CON groups. **E** KEGG pathway analysis enriched form all differential metabolites. **F** Correlation heatmap between top 20 abundance of bacteria and top 20 differential metabolites (*P* < 0.068). PLS-DA, Partial least squares discrimination analysis; OPLS-DA, Orthogonal partial least squares-discriminant analysis

As shown in Fig. 7, the PLS-DA score plot and OPLS-DA score plot both showed a clear separation of metabolic profiles between the CON and SIL groups (Fig. 7A and B). The metabolomic analysis identified a total of 366 metabolites (Fig. 7C). In SIL group, we identified 9 differential metabolites compared with CON group (5 in positive ion mode and 4 in negative ion mode; Fig. 7C). The content of ricinoleic acid, thiamine monophosphate, glucosamine-6-phosphate, hydroxyproline and *cis*-4-Hydroxy-D-proline were significantly higher in SIL group than CON group, but uridine 5′-diphosphate (UDP), N2,N2-dimethylguanosine, vitamin B_7_ and ketoleucine concentrations were lower in SIL group than CON group (Fig. 7D). A further KEGG enrichment analysis showed that the different metabolites were closely related to valine, leucine and isoleucine biosynthesis, biotin metabolism, alanine, aspartate and glutamate metabolism, thiamine metabolism, arginine proline metabolism and the ATP-binding cassette (ABC) transporters pathway. Arginine proline metabolism and ABC transporters were the most significant (Fig. 7E).

## Discussion

Enhancing the production performance and egg quality of late-stage laying hens is crucial for increasing their economic value [29]. The liver, the crucial organ in lipid metabolism [5], is particularly susceptible to lipid accumulation and oxidative stress during this period [1]. These conditions can adversely affect the production performance of the hens. Previous studies have indicated that dietary supplementation with silymarin not only improves the laying rate and egg weight but also reduces the feed-to-egg ratio [23]. Similar improvements in production performance have been observed in broilers when silymarin is added to their diets [20, 30]. Recent research supports these findings, showing that 500 mg/kg of dietary silymarin significantly enhances average egg weight and lowers the feed-to-egg ratio, aligning with earlier reports. Eggshell strength and thickness are critical indicators of egg quality. Nie et al. [31] demonstrated that eggshell quality is linked to the absorption of calcium ions in the small intestine of laying hens. In this study, the addition of 500 mg/kg of silymarin appeared to enhance eggshell thickness and strength, likely by improving intestinal absorption capabilities [32].

Interestingly, we observed a significant decrease in the laying rate of the CON group as the experiment progressed. In contrast, the laying rates in the SIL groups remained stable, suggesting that silymarin mitigates the decline in laying rates among late-stage laying hens. The liver, crucial for lipid metabolism and yolk precursor production [33], often exhibits health through the levels of transaminases such as ALT and AST in hepatocytes [34]. This study found that a dietary addition of 500 mg/kg silymarin reduced serum ALT and AST levels by week 12. Consistent with our findings, previous studies reported reductions in serum ALT and AST levels in broilers exposed to mycotoxins [16] and in models of chemically induced liver damage [35].

Excessive fat accumulation in hepatocytes can lead to histological changes in the liver. Serum TG and TC levels were important indexes to explain the capacity of lipid metabolism in animals. In our experiment, SIL treatment significantly lowered serum TG and TC level. These results align with findings by Ghazaghi et al. [36], who noted a significant decrease in serum TC in quails treated with silymarin, and a recent study showing reduced serum TC and LDL-C in broilers [20]. Moreover, HDL and LDL play essential roles in hepatic lipid homeostasis [37]. Similar to previous studies [38, 39], our research found that silymarin supplementation decreased serum LDL-C and HDL-C levels in late laying hens by week 12. Specifically, we observed an increase in serum VLDL levels, which are critical for transporting endogenous triglycerides from the liver to tissues [40]. Further investigation into liver expressions of ApoB, ApoVLDLII, VTG, and GPR30 which are involved in VLDL assembly and yolk precursor transport [41, 42] revealed that silymarin enhances VLDL assembly and yolk precursor synthesis. These findings suggest that silymarin could improve the production performance of late-stage laying hens by alleviating liver steatosis and enhancing the synthesis of lipoproteins and yolk precursors. In this context, a 500 mg/kg dose of SIL was the most effective in regulating lipid metabolism in these hens.

To further elucidate the underlying mechanisms of silymarin on lipid metabolism in laying hens, we analyzed the expression of genes related to lipid metabolism, including *ACC*, *FASN*, *PPAR-α*, and *SREBP-1* [38]. The ELOVL family, particularly *ELOVL6* and *ELOVL7*, are known to respond to lipid deposition in broilers [43]. Our findings show that silymarin enhances the expression of these genes, suggesting a beneficial role in the elongation of very long chain fatty acids. Consistent with our study, previous research has indicated that silymarin and silybin can suppress genes involved in lipid synthesis, such as *FASN* and *SCD* [44]. Furthermore, silymarin has been shown to increase the expression of lipolysis-related genes such as *ACC* [45] and to prevent lipid accumulation by modulating *SREBP-1c* in diabetic rats [38]. Despite clarifying the regulatory effects of silymarin on lipid metabolism-related genes in the liver, the upstream factors remain less understood. Estrogen is known to significantly influence lipid metabolism [46, 47], and its reduced levels and receptor expression in aging hens correlate with decreased yolk precursor formation and laying rate [48, 49]. Studies, including one by Quarantelli et al. [50], have shown that dietary silymarin enhances ovarian activity, possibly through increased estradiol-17-β and progesterone levels in follicles. Our study extends these findings, indicating that silymarin modulates estrogen receptor expressions (upregulating *ERβ* and downregulating *ERα*) in hen livers, which may explain the estrogenic effects observed in other studies, such as those by Eisa et al. [51] and Sánchez et al. [52].

Another important factor that participated in liver lipid metabolism is bile acid metabolism. The bile acid receptor *FXR* belongs to the family of ligand-activated transcription factors and can negatively regulate the bile acid synthesis rate-limiting enzyme *CYP7A1* [53]. In chicken primary hepatocytes, activating *FXR* could regulate bile acid and lipid metabolism [54]. Another research highlighted the ability of silymarin to activate *FXR* in HepG2 cells, thus leading to a downregulation of *CYP7A1* gene expression [55]. Additionally, *BSEP* is responsible for pumping bile acids produced in the liver against an adverse concentration gradient into the bile canaliculi [56]. *MRP2* is the major bile acid transport protein in liver, which may promote bile acid transport to the intestine [57]. The previous study indicated that silymarin plays a vital role in biliary excretion of *MRP2* [58]. Silymarin also could prevent cholestasis-associated recovery of the *BSEP* [59]. Similar with above results, our results showed that silymarin upregulated *FXR* expression and downregulated the *CYP7A1* expression. In our study, silymarin reduced the endogenous bile acid synthesis and accelerate the enterohepatic circulation.

Cecal microbiota has been shown to correlate with laying performance of layers [60, 61]. Lucke et al. [62] reported that the most dominant bacteria at the phylum level were Bacteroidota and Firmicutes in poultry which was consistent with our results. Silymarin inhibited the growth of pathogenic bacteria and promoted the colonization of beneficial bacteria in the intestine of poultry [63, 64]. Lipid metabolism of poultry was related to gut microbiota [65, 66]. The LefSe analysis revealed that the dominant bacteria in the SIL group were primarily associated with the lipid metabolism of the host, including *s_Phocaeicola s*_*Limosilactobacillus_sp012843675, s_Pelethomonas_sp017887695,* and s_*Flavonifractor_avistercoris*. Zhou et al. [67] reported that *Limosilactobacillus-reuteri* improved lipid metabolism via regulating gut microbiota in mice fed with a High-Fat diet. Previous studies reported that dietary supplementation with *Limosilactobacillus* improved performance production in poultry [68, 69]. Cristina et al. [70] found that NAFLD decreased the abundance of *Flavonifractor* in human intestine. Mikami et al. [71] found that oral *Flavonifractor* could attenuate inflammatory responses in obese adipose tissue. In our study, SIL group increase the abundance of *Flavonifractor,* which had a potential role in promoting lipid metabolism The above experiments demonstrated that the regulation of lipid metabolism by silymarin in late laying hens was inextricably linked to its modulation of intestinal microbial composition.

Cecal microbiota has been reported to participate in bile acid metabolism. Previous study showed that silymarin could regulate bile acid metabolism via *FXR* [72]. In our study, silymarin improved the relative abundance of *Phocaeicola* genus, which was positively correlated with unconjugated chenodeoxycholic acid and performed bile acid deconjugation [73, 74]. Ye et al. [74] reported that *Lactobacillus* regulated bile acid metabolism via regulating FXR signaling-mediated. Furthermore, the functional unit analysis revealed that silymarin increased the expression of microbial gene related to secondary bile acid metabolism. The results demonstrated that silymarin alleviated bile acid stagnation in the body by modulating gut microbial function and enhancing the ability of metabolic microorganisms to metabolize bile acids.

Through the metabolomics analysis, we found 5 significantly up-regulating metabolites in SIL treatment, including ricinoleic acid, thiamine monophosphate, Hydroxyproline, *cis*-4-hydroxy-D-proline and glucosamine 6-phosphate. Biotin could act as a coenzyme for carboxylases regulating lipid and amino acid metabolism [75]. Oloyo et al. [76] reported that dietary addition of biotin reduced serum TG, TC contents in chicks. The results of this study showed that differential metabolites biotin metabolism, which implied that biotin may contribute to the regulation of lipid metabolism by silymarin. Recent study showed that high-dose thiamine could prevent the development of experimental fatty liver driven by overnutrition [77]. Hamano et al. [78] reported that dietary thiamine supplementation reduced plasma TG in broiler chicks.

Our results showed that TMP content was significantly higher in SIL group. Therefore, it can be inferred that silymarin may enhance the absorption of thiamine by the host. In bacteria, ABC importers were involved in the uptake of nutrients and micro-nutrients through medium- and high-affinity pathways [79], which promoted cellular nutrient uptake of microorganisms and host. The results of differential metabolite KEGG pathway enrichment analysis showed significant upregulation of ABC transporters in the SIL group. These were considered to elucidate the effect of silymarin on lipid and bile acid metabolism by regulating intestinal microorganisms and their metabolites.

## Conclusions

In summary, silymarin supplementation could improve performance by regulating liver lipid metabolism, estrogen receptors activity and improving cecal microbiota and its function in late laying hens. Specifically, supplementation with silymarin decreased serum TG, TC, HDL-C, LDL-C content, and increased serum VLDL content by regulating the expression of *FASN*, *ACC*, *SCD*, *PPAR-α* and *ApoVLDLII *in liver. Moreover, dietary silymarin regulated the expression of *FXR*, *CYP7A1*, *BSEP* and *MRP2* in liver and altered the cecal microbiological structure and three species of *Phocaeicola* were dominated microbial functions which were enriched in secondary bile acid synthesis. The results highlight that silymarin is a feasible feed additive for late laying hens, with the optimal dosage being 500 mg/kg. The present study provided a theoretical basis for the application of silymarin extract in late laying hens.

## Supplementary Information

**Additional file 1:** **Fig. S1.** Ingredient testing of SIL. **Table S1.** Average laying rate (pre-feeding period).

## Abbreviations

ApoVLDLII: Apolipoprotein-VLDLII
ADFI: Average daily feed intake
ALT: Alanine aminotransferase
AST: Aspartate aminotransferase
ANOVA: One-way analysis of variance
ACC: Acetyl-CoA carboxylase
ApoB: Apolipoprotein B
ABC: ATP-binding cassette
BSEP: Bile salt export pump
CYP7A1: Cholesterol 7 alpha-monooxygenase
ERα: Estrogen receptorα
ELOVL6: Elongation of very long chain fatty acids 6
ELOVL7: Elongation of very long chain fatty acids 7
ESRα: Estrogen receptor α
ESRβ: Estrogen receptor β
FASN: Fatty acid synthase
FXR: Farnesoid X receptor
GPR30: G protein-coupled estrogen receptor 30
HDL-C: High-density lipoprotein-cholesterol
LDL-C: Low-density lipoprotein-cholesterol
LEfSe: Linear discriminant analysis effect size
MRP2: Multidrug resistance associated-protein
NLRP3: NLR family pyrin domain containing 3
NMDS: Nonmetric multidimensional scaling
OPLS-DA: Orthogonal partial least squares discriminant analysis
PCoA: Principal coordinate analysis
PCA: Principal component analysis
PPAR-α: Peroxisome proliferators-activated receptor-α
SIL: Silymarin
SD: Standard deviation
SCD: Stearoyl-CoA desaturase
SREBP-1: Sterol-regulatory element binding protein-1
TG: Triglyceride
TC: Total cholesterol
VLDL: Very low-density lipoprotein
VTG: Vitellogenesis

## References

[1] Li H, Wang T, Xu C, Wang D, Ren J, Li Y, et al. Transcriptome profile of liver at different physiological stages reveals potential mode for lipid metabolism in laying hens. BMC Genomics. 2015;16:763. 10.1186/s12864-015-1943-0. 26452545 10.1186/s12864-015-1943-0 PMC4600267

[2] Zhang H, Shen L, Li Y, Xu Z, Zhang X, Yu J, et al. Genome-wide association study for plasma very low-density lipoprotein concentration in chicken. J Anim Breed Genet. 2019;136(5):351–61. 10.1111/jbg.12397. 31037768 10.1111/jbg.12397

[3] Schneider WJ, Carroll R, Severson DL, Nimpf J. Apolipoprotein vldl-ii inhibits lipolysis of triglyceride-rich lipoproteins in the laying hen. J Lipid Res. 1990;31(3):507–13. 2111369

[4] Shini A, Shini S, Bryden WL. Fatty liver haemorrhagic syndrome occurrence in laying hens: impact of production system. Avian Pathol. 2019;48(1):25–34. 10.1080/03079457.2018.1538550. 30345810 10.1080/03079457.2018.1538550

[5] Zaefarian F, Abdollahi M, Cowieson A, Ravindran V. Avian liver: the forgotten organ. Animals. 2019;9(2):63. 10.3390/ani9020063. 30781411 10.3390/ani9020063 PMC6406855

[6] Han GP, Kim DY, Kim KH, Kim JH, Kil DY. Effect of dietary concentrations of metabolizable energy and neutral detergent fiber on productive performance, egg quality, fatty liver incidence, and hepatic fatty acid metabolism in aged laying hens. Poult Sci. 2023;102(4):102497. 10.1016/j.psj.2023.102497. 36739800 10.1016/j.psj.2023.102497 PMC9932556

[7] Cui Z, Amevor FK, Feng Q, Kang X, Song W, Zhu Q, et al. Sexual maturity promotes yolk precursor synthesis and follicle development in hens via liver-blood-ovary signal axis. Animals. 2020;10(12):2348. 10.3390/ani10122348. 33317071 10.3390/ani10122348 PMC7763865

[8] Qiang T, Wang J, Ding X, Zeng Q, Bai S, Lv L, et al. The improving effect of soybean isoflavones on ovarian function in older laying hens. Poult Sci. 2023;102(10):102944. 10.1016/j.psj.2023.102944. 37531725 10.1016/j.psj.2023.102944 PMC10407823

[9] Zhang L, Zhong G, Gu W, Yin N, Chen L, Shi S. Dietary supplementation with daidzein and Chinese herbs, independently and combined, improves laying performance, egg quality and plasma hormone levels of post-peak laying hens. Poult Sci. 2021;100(6):101115. 10.1016/j.psj.2021.101115. 33975040 10.1016/j.psj.2021.101115 PMC8131741

[10] Amevor FK, Cui Z, Ning Z, Du X, Jin N, Shu G, et al. Synergistic effects of quercetin and vitamin e on egg production, egg quality, and immunity in aging breeder hens. Poult Sci. 2021;100(12):101481. 10.1016/j.psj.2021.101481 . 34717121 10.1016/j.psj.2021.101481 PMC8564671

[11] Gao X, Liu S, Ding C, Miao Y, Gao Z, Li M, et al. Comparative effects of genistein and bisphenol a on non-alcoholic fatty liver disease in laying hens. Environ Pollut. 2021;288:117795. 10.1016/j.envpol.2021.117795. 34274649 10.1016/j.envpol.2021.117795

[12] Li H, Hou Y, Hu J, Li J, Liang Y, Lu Y, et al. Dietary naringin supplementation on hepatic yolk precursors formation and antioxidant capacity of three-yellow breeder hens during the late laying period. Poult Sci. 2023;102(5):102605. 10.1016/j.psj.2023.102605. 36940650 10.1016/j.psj.2023.102605 PMC10033312

[13] Liu Y, Wang Y, Wang C, Sun X, Gao S, Liu R, et al. Alterations in hepatic transcriptome and cecum microbiota underlying potential ways to prevent early fatty liver in laying hens. Poult Sci. 2023;102(5):102593. 10.1016/j.psj.2023.102593. 36972673 10.1016/j.psj.2023.102593 PMC10066560

[14] Lv Z, Fan H, Zhang B, Ning C, Xing K, Guo Y. Dietary genistein supplementation in laying broiler breeder hens alters the development and metabolism of offspring embryos as revealed by hepatic transcriptome analysis. FASEB J. 2018;32(8):4214–28. 10.1096/fj.201701457R. 29518347 10.1096/fj.201701457R

[15] Gillessen A, Schmidt HH. Silymarin as supportive treatment in liver diseases: a narrative review. Adv Ther. 2020;37(4):1279–301. 10.1007/s12325-020-01251-y. 32065376 10.1007/s12325-020-01251-y PMC7140758

[16] Armanini EH, Boiago MM, Cécere BGDO, Oliveira PV, Teixeira CJS, Strapazzon JV, et al. Protective effects of silymarin in broiler feed contaminated by mycotoxins: growth performance, meat antioxidant status, and fatty acid profiles. Trop Anim Health Pro. 2021;53(4):442. 10.1007/s11250-021-02873-2. 10.1007/s11250-021-02873-2 34410508

[17] Khatoon A, Zargham Khan M, Khan A, Saleemi MK, Javed I. Amelioration of ochratoxin a-induced immunotoxic effects by silymarin and vitamin e in white leghorn cockerels. J Immunotoxicol. 2012;10(1):25–31. 10.3109/1547691X.2012.686533. 22734832 10.3109/1547691X.2012.686533

[18] Elnesr SS, Elwan HAM, El Sabry MI, Shehata AM. The nutritional importance of milk thistle (silybum marianum) and its beneficial influence on poultry. World’s Poult Sci J. 2023;79(4):751–68. 10.1080/00439339.2023.2234339.

[19] Seidlová-Wuttke D, Becker T, Christoffel V, Jarry H, Wuttke W. Silymarin is a selective estrogen receptor β (erβ) agonist and has estrogenic effects in the metaphysis of the femur but no or antiestrogenic effects in the uterus of ovariectomized (ovx) rats. J Steroid Biochem Mol Biol. 2003;86(2):179–88. 10.1016/S0960-0760(03)00270-X. 14568570 10.1016/s0960-0760(03)00270-x

[20] Zhou ZB, Ding YN, Bai XL, Song ZH, He X, Liu ZK. Effects of silymarin on growth performance, slaughter performance, lipid metabolism and bile acid metabolism related gene expression of fast growth yellow feather broilers. Chin J Anim Nutr. 2022;34(08):5000–10.

[21] Zhou ZB, Wang WW, Xu YD, Lai YQ, Bai XL, Song ZH, et al. Effects of silymarin on antioxidant capacity, immune function and intestinal flora of rapid tallow feather broilers. Chin J Anim Nutr. 2022;34(12):7686–700.

[22] Faryadi S, Sheikhahmadi A, Farhadi A, Nourbakhsh H. Effects of silymarin and nano-silymarin on performance, egg quality, nutrient digestibility, and intestinal morphology of laying hens during storage. Ital J Anim Sci. 2021;20(1):1633–44. 10.1080/1828051X.2021.1975503.

[23] Sun W, Hua S, Li X, Shen L, Wu H, Ji H. Microbially produced vitamin b12 contributes to the lipid-lowering effect of silymarin. Nat Commun. 2023;14(1):477. 10.1038/s41467-023-36079-x. 36717576 10.1038/s41467-023-36079-x PMC9887073

[24] Kechin A, Boyarskikh U, Kel A, Filipenko M. Cutprimers: a new tool for accurate cutting of primers from reads of targeted next generation sequencing. J Comput Biol. 2017;24(11):1138–43. 10.1089/cmb.2017.0096. 28715235 10.1089/cmb.2017.0096

[25] Chen S, Zhou Y, Chen Y, Gu J. Fastp: an ultra-fast all-in-one fastq preprocessor. Bioinformatics. 2018;34(17):i884–890. 10.1093/bioinformatics/bty560. 30423086 10.1093/bioinformatics/bty560 PMC6129281

[26] Wood DE, Lu J, Langmead B. Improved metagenomic analysis with kraken 2. Genome Biol. 2019;20(1):257. 10.1186/s13059-019-1891-0. 31779668 10.1186/s13059-019-1891-0 PMC6883579

[27] Steinegger M, Söding J. Mmseqs2 enables sensitive protein sequence searching for the analysis of massive data sets. Nat Biotechnol. 2017;35(11):1026–8. 10.1038/nbt.3988. 29035372 10.1038/nbt.3988

[28] Patro R, Duggal G, Love MI, Irizarry RA, Kingsford C. Salmon provides fast and bias-aware quantification of transcript expression. Nat Methods. 2017;14(4):417–9. 10.1038/nmeth.4197. 28263959 10.1038/nmeth.4197 PMC5600148

[29] Dai D, Wu S, Zhang H, Qi G, Wang J. Dynamic alterations in early intestinal development, microbiota and metabolome induced by in ovo feeding of l-arginine in a layer chick model. J Anim Sci Biotechnol. 2020;11(1):19. 10.1186/s40104-020-0427-5. 32175081 10.1186/s40104-020-0427-5 PMC7063725

[30] Barreiro Carpio M, Valdes-Pena MA, Molina DA, Espinoza Cabello SEJ, Sialer Guerrero CA, Cribillero G, et al. Evaluation of commercial doses of a feed additive and silymarin on broiler performance with and without ccl4-induced liver damage. Poul Sci. 2024;103(5):103567. 10.1016/j.psj.2024.103567. 10.1016/j.psj.2024.103567 PMC10909905 38417302

[31] Nie W, Yang Y, Yuan J, Wang Z, Guo Y. Effect of dietary nonphytate phosphorus on laying performance and small intestinal epithelial phosphate transporter expression in dwarf pink-shell laying hens. J Anim Sci Biotechnol. 2013;4:34. 10.1186/2049-1891-4-34. 10.1186/2049-1891-4-34 PMC3849798 24028402

[32] Jahanian E, Mahdavi AH, Asgary S, Jahanian R. Effects of dietary inclusion of silymarin on performance, intestinal morphology and ileal bacterial count in aflatoxin-challenged broiler chicks. J Anim Physiol N. 2017;101(5):e43–54. 10.1111/jpn.12556. 10.1111/jpn.12556 28052409

[33] Schneider WJ. Lipoprotein receptors in oocyte growth. Clin Investig. 1992;70(5):385–90. 10.1007/BF00235517. 1318130 10.1007/BF00235517

[34] Guerrini A, Tedesco DEA. Restoring activity of milk thistle (*Silybum marianum* L.) on serum biochemical parameters, oxidative status, immunity, and performance in poultry and other animal species, poisoned by mycotoxins: a review. Animals. 2023;13(3):330. 10.3390/ani13030330. 10.3390/ani13030330 PMC9913068 36766219

[35] Baradaran A, Samadi F, Ramezanpour SS, Yousefdoust S. Hepatoprotective effects of silymarin on CCl_4_-induced hepatic damage in broiler chickens model. Toxicol Rep. 2019;6:788–94. 10.1016/j.toxrep.2019.07.011. 10.1016/j.toxrep.2019.07.011 PMC6698800 31440455

[36] Ghazaghi M, Isazaei A, Bagherzadeh-Kasmani F, Mehri M. Regression-derived optimal milk thistle in growing quail’s diet. Poult Sci. 2024;103(3):103465. 10.1016/j.psj.2024.103465. 38277889 10.1016/j.psj.2024.103465 PMC10840340

[37] Jiang X, Zhang B, Lan F, Zhong C, Jin J, Li X, et al. Host genetics and gut microbiota jointly regulate blood biochemical indicators in chickens. Appl Microbiol Biot. 2023;107(24):7601–20. 10.1007/s00253-023-12814-8. 10.1007/s00253-023-12814-8 PMC10656342 37792060

[38] Kheiripour N, Karimi J, Khodadadi I, Tavilani H, Goodarzi MT, Hashemnia M. Silymarin prevents lipid accumulation in the liver of rats with type 2 diabetes via sirtuin1 and SREBP-1c. J Basic Clin Physiol Pharmacol. 2018;29(3):301–8. 10.1515/jbcpp-2017-0122. 10.1515/jbcpp-2017-0122 29476664

[39] Zaker Esteghamati H, Seidavi A, Bouyeh M. The effects ofcynara scolymus andsilybum marianum on growth, carcass and organ characteristics, immunity, blood constitutes, liver enzymes, jejunum morphology, and fatty acid profile of breast meat in broilers. Food Sci Nutr. 2021;9(12):6692–706. 10.1002/fsn3.2620. 34925799 10.1002/fsn3.2620 PMC8645736

[40] Walzem RL, Hansen RJ, Williams DL, Hamilton RL. Estrogen induction of vldly assembly in egg-laying hens. J Nutr. 1999;129(2S Suppl):467S–472S. 10.1093/jn/129.2.467S. 10064311 10.1093/jn/129.2.467S

[41] Schneider WJ. Yolk precursor transport in the laying hen. Curr Opin Lipidol. 1995;6(2):92–6. 10.1097/00041433-199504000-00006. 7773574 10.1097/00041433-199504000-00006

[42] Walzem RL, Davis PA, Hansen RJ. Overfeeding increases very low density lipoprotein diameter and causes the appearance of a unique lipoprotein particle in association with failed yolk deposition. J Lipid Res. 1994;35(8):1354–66. 7989860

[43] Wang D, Li X, Zhang P, Cao Y, Zhang K, Qin P, et al. Elovl gene family plays a virtual role in response to breeding selection and lipid deposition in different tissues in chicken (Gallus gallus). BMC Genomics. 2022;23:705. 10.1186/s12864-022-08932-8. 10.1186/s12864-022-08932-8 PMC9575239 36253734

[44] Suh HJ, Cho SY, Kim EY, Choi H. Blockade of lipid accumulation by silibinin in adipocytes and zebrafish. Chem-Biol Interact. 2015;227:53–62. 10.1016/j.cbi.2014.12.027. 25559859 10.1016/j.cbi.2014.12.027

[45] Xiao P, Ji H, Ye Y, Zhang B, Chen Y, Tian J, et al. Dietary silymarin supplementation promotes growth performance and improves lipid metabolism and health status in grass carp (*Ctenopharyngodon idellus*) fed diets with elevated lipid levels. Fish Physiol Biochem. 2017;43:245–63. 10.1007/s10695-016-0283-6. 10.1007/s10695-016-0283-6 27632016

[46] Ren J, Tian W, Jiang K, Wang Z, Wang D, Li Z, et al. Global investigation of estrogen-responsive genes regulating lipid metabolism in the liver of laying hens. BMC Genomics. 2021;22:428. 10.1186/s12864-021-07679-y. 10.1186/s12864-021-07679-y PMC8190866 34107898

[47] Jiang Z, Yang Z, Zhang H, Yao Y, Ma H. Genistein activated adenosine 5’-monophosphate-activated protein kinase-sirtuin1/peroxisome proliferator-activated receptor γ coactivator-1α pathway potentially through adiponectin and estrogen receptor β signaling to suppress fat deposition in broiler chickens. Poult Sci. 2021;100(1):246–55. 10.1016/j.psj.2020.10.013. 33357687 10.1016/j.psj.2020.10.013 PMC7772704

[48] Gu YF, Chen YP, Jin R, Wang C, Wen C, Zhou YM. Age-related changes in liver metabolism and antioxidant capacity of laying hens. Poult Sci. 2021;100(12):101478. 10.1016/j.psj.2021.101478. 34695635 10.1016/j.psj.2021.101478 PMC8554276

[49] Hansen KK, Kittok RJ, Sarath G, Toombs CF, Caceres N, Beck MM. Estrogen receptor-alpha populations change with age in commercial laying hens. Poult Sci. 2003;82(10):1624–9. 10.1093/ps/82.10.1624. 14601742 10.1093/ps/82.10.1624

[50] Quarantelli A, Romanelli S, Basini G, Righi F. The effects of silymarin on ovarian activity and productivity of laying hens. Ital J Anim Sci. 2009;8(sup2):769–71. 10.4081/ijas.2009.s2.769.

[51] Eisa MA, Mansour AM, Salama SA, Elsadek BEM, Ashour AA, Abdelghany TM. Estrogen/estrogen receptor activation protects against DEN-induced liver fibrosis in female rats via modulating TLR-4/NF-kβ signaling. Eur J Pharmacol. 2023;960:176165. 10.1016/j.ejphar.2023.176165. 10.1016/j.ejphar.2023.176165 38059444

[52] Sánchez GJ, Santiago LD. Action of silymarin on the fatty acid composition of the hepatic lipids of estrogenized chickens. Arch Farmacol Toxicol. 1981;7(1):143–50. 7325707

[53] Sun L, Xin Q, Jiao H, Wang X, Zhao J, Li H, et al. Effect of exogenous bile salts supplementation on the performance and hepatic lipid metabolism of aged laying hens. J Anim Sci. 2023;101:skad334. 10.1093/jas/skad334. 10.1093/jas/skad334 PMC11025372 37773415

[54] Sato K, Kamada T. Regulation of bile acid, cholesterol, and fatty acid synthesis in chicken primary hepatocytes by different concentrations of T0901317, an agonist of liver x receptors. Comp Biochem Physiol Mol Integr Physiol. 2011;158(2):201–6. 10.1016/j.cbpa.2010.10.028. 10.1016/j.cbpa.2010.10.028 21056113

[55] Gu M, Zhao P, Huang J, Zhao Y, Wang Y, Li Y, et al. Silymarin ameliorates metabolic dysfunction associated with diet-induced obesity via activation of farnesyl X receptor. Front Pharmacol. 2016;7:345. 10.3389/fphar.2016.00345. 10.3389/fphar.2016.00345 PMC5039206 27733832

[56] Zhang Y, Chen H, Cong W, Zhang K, Jia Y, Wu L. Chronic heat stress affects bile acid profile and gut microbiota in broilers. Int J Mol Sci. 2023;24(12):10238. 10.3390/ijms241210238. 37373380 10.3390/ijms241210238 PMC10299590

[57] Osselaere A, Li SJ, De Bock L, Devreese M, Goossens J, Vandenbroucke V, et al. Toxic effects of dietary exposure to T-2 toxin on intestinal and hepatic biotransformation enzymes and drug transporter systems in broiler chickens. Food Chem Toxicol. 2013;55:150–5. 10.1016/j.fct.2012.12.055. 10.1016/j.fct.2012.12.055 23313610

[58] Miranda SR, Lee JK, Brouwer KL, Wen Z, Smith PC, Hawke RL. Hepatic metabolism and biliary excretion of silymarin flavonolignans in isolated perfused rat livers: role of multidrug resistance-associated protein 2 (ABcc2). Drug Metab Dispos. 2008;36(11):2219–26. 10.1124/dmd.108.021790. 10.1124/dmd.108.021790 PMC2660567 18687803

[59] Crocenzi FA, Basiglio CL, Pérez LM, Portesio MS, Pozzi EJS, Roma MG. Silibinin prevents cholestasis-associated retrieval of the bile salt export pump, bsep, in isolated rat hepatocyte couplets: possible involvement of camp. Biochem Pharmacol. 2005;69(7):1113–20. 10.1016/j.bcp.2005.01.009. 15763547 10.1016/j.bcp.2005.01.009

[60] Wu G, Li Z, Zheng Y, Zhang Y, Liu L, Gong D, et al. Supplementing cholamine to diet lowers laying rate by promoting liver fat deposition and altering intestinal microflora in laying hens. Poult Sci. 2022;101(10):102084. 10.1016/j.psj.2022.102084. 36055021 10.1016/j.psj.2022.102084 PMC9449860

[61] Liu M, Kang Z, Cao X, Jiao H, Wang X, Zhao J, et al. Prevotella and succinate treatments altered gut microbiota, increased laying performance, and suppressed hepatic lipid accumulation in laying hens. J Anim Sci Biotechnol. 2024;15:26. 10.1186/s40104-023-00975-5. 10.1186/s40104-023-00975-5 PMC10874536 38369510

[62] Lucke A, Böhm J, Zebeli Q, Metzler-Zebeli BU. Dietary deoxynivalenol contamination and oral lipopolysaccharide challenge alters the cecal microbiota of broiler chickens. Front Microbiol. 2018;9(804):804. 10.3389/fmicb.2018.00804. 29922239 10.3389/fmicb.2018.00804 PMC5996912

[63] Jahanian E, Mahdavi AH, Jahanian R. Silymarin improved the growth performance via modulating the microbiota and mucosal immunity in escherichia coli-challenged broiler chicks. J Livest Sci. 2021;249;104529. 10.1016/j.livsci.2021.104529.

[64] Kareem SM, Mahmood SS, Hindi NK. Effects of curcumin and silymarin on the shigella dysenteriae and campylobacter jejuni in vitro. J Gastrointest Canc. 2020;51(3):824–8. 10.1007/s12029-019-00301-1. 10.1007/s12029-019-00301-1 31482407

[65] Fu Q, Wang P, Zhang Y, Wu T, Huang J, Song Z. Effects of dietary inclusion of asiaticoside on growth performance, lipid metabolism, and gut microbiota in yellow-feathered chickens. Animals (Basel). 2023;13(16):2653. 10.3390/ani13162653. 10.3390/ani13162653 PMC10451259 37627444

[66] Shen Y, Zhang S, Zhao X, Shi S. Evaluation of a lecithin supplementation on growth performance, meat quality, lipid metabolism, and cecum microbiota of broilers. Animals (Basel). 2021;11(9):2537. 10.3390/ani11092537. 34573503 10.3390/ani11092537 PMC8465824

[67] Yang Z, Zhu X, Wen A, Ran J, Qin L, Zhu Y. Coix seed-based milk fermented with limosilactobacillus reuteri improves lipid metabolism and gut microbiota in mice fed with a high-fat diet. Front Nutri (Lausanne). 2022;9(9):921255. 10.3389/fnut.2022.921255. 10.3389/fnut.2022.921255 PMC9320324 35903451

[68] Wan Z, Sun N, Luo M, Gan B, Yao Z, Cao X, et al. Promotion of egg production rate and quality using *Limosilactobacillus oris* BSLO 1801, a potential probiotic screened from feces of laying hens with higher egg productive performance. Probiotics Antimicro. 2023;15(3):535–47. 10.1007/s12602-021-09856-7. 10.1007/s12602-021-09856-7 34697775

[69] Hati S, Ramanuj K, Basaiawmoit B, Koringa P, Desai M, Ghodasara DJ et al. Significance of limosilactobacillus fermentum and saccharomyces cerevisiae on the growth performance, haematological traits, serum biochemistry, faecal and caeca microbiota of broiler chickens. J Am Nutr Assoc. 2023:706–25. 10.1080/27697061.2022.2149634. 10.1080/27697061.2022.2149634 36449022

[70] Rodriguez-Diaz C, Taminiau B, Garcia-Garcia A, Cueto A, Robles-Diaz M, Ortega-Alonso A, et al. Microbiota diversity in nonalcoholic fatty liver disease and in drug-induced liver injury. Pharmacol Res. 2022;182:106348. 10.1016/j.phrs.2022.106348. 35817360 10.1016/j.phrs.2022.106348

[71] Mikami A, Ogita T, Namai F, Shigemori S, Sato T, Shimosato T. Oral administration of flavonifractor plautii attenuates inflammatory responses in obese adipose tissue. Mol Biol Rep. 2020;47(9):6717–25. 10.1007/s11033-020-05727-6. 32808115 10.1007/s11033-020-05727-6

[72] Li Y, Tian Y, Cai W, Wang Q, Chang Y, Sun Y, et al. Novel ι-carrageenan tetrasaccharide alleviates liver lipid accumulation via the bile acid-fxr-shp/pxr pathway to regulate cholesterol conversion and fatty acid metabolism in insulin-resistant mice. J Agric Food Chem. 2021;69(34):9813–21. 10.1021/acs.jafc.1c04035. 34415766 10.1021/acs.jafc.1c04035

[73] Noh C, Jung W, Yang MJ, Kim WH, Hwang JC. Alteration of the fecal microbiome in patients with cholecystectomy: potential relationship with postcholecystectomy diarrhea - before and after study. Int J Surg. 2023;109(9):2585–97. 10.1097/JS9.0000000000000518. 10.1097/JS9.0000000000000518 PMC10498850 37288587

[74] Ye X, Huang D, Dong Z, Wang X, Ning M, Xia J, et al. Fxr signaling-mediated bile acid metabolism is critical for alleviation of cholesterol gallstones bylactobacillus strains. Microbiol Spectr. 2022;10(5):e00518–22. 10.1128/spectrum.00518-22. 36036629 10.1128/spectrum.00518-22 PMC9603329

[75] Järvinen E, Ismail K, Muniandy M, Bogl LH, Heinonen S, Tummers M, et al. Biotin-dependent functions in adiposity: a study of monozygotic twin pairs. Int J Obes. 2016;40(5):788–95. 10.1038/ijo.2015.237. 10.1038/ijo.2015.237 26601567

[76] Oloyo RA, Ogunmodede BK. Preliminary investigation on the effect of dietary supplemental biotin and palm kernel oil on blood, liver and kidney lipids in chicks. Arch Tierernahr. 1992;42(3–4):263–72. 10.1080/17450399209428540. 1296556 10.1080/17450399209428540

[77] Kalyesubula M, Mopuri R, Asiku J, Rosov A, Yosefi S, Edery N, et al. High-dose vitamin B1 therapy prevents the development of experimental fatty liver driven by overnutrition. Dis models Mech. 2021;14(3):dmm048355. 10.1242/dmm.048355. 10.1242/dmm.048355 PMC7988776 33608323

[78] Hamano Y, Okada S, Tanaka T. Effects of thiamine and clenbuterol on body composition, plasma metabolites and hepatic oxygen consumption in broiler chicks. Br Poult Sci. 1999;40(1):127–30. 10.1080/00071669987953. 10405048 10.1080/00071669987953

[79] Sun H, Xu W, Gu T, Sun J, Li C, Chen L, et al. Association of residual feed intake with intestinal microbiome and metabolome in laying period of ducks. Front Microbiol. 2023;14:1138914. 10.3389/fmicb.2023.1138914. 37250027 10.3389/fmicb.2023.1138914 PMC10213451

