# Correction: Dietary silymarin improves performance by altering hepatic lipid metabolism and cecal microbiota function and its metabolites in late laying hens

**DOI:** 10.1186/s40104-025-01161-5

**Published:** 2025-02-14

**Authors:** Yanghao Guo, Yudong Xu, Derun Wang, Shihao Yang, Zehe Song, Rui Li, Xi He

**Affiliations:** 1 College of Animal Science and Technology, Hunan Agricultural University, Changsha, Hunan 410128 China; 2 Hunan Engineering Research Center of Poultry Production Safety, Changsha, Hunan 410128 China; 3 Yuelushan Laboratory, Hunan Agricultural University, Changsha, Hunan 410128 China; 4 Institute of Subtropical Agriculture, Chinese Academy of Sciences, Changsha, Hunan 410125 China

**Correction** **:** **J Animal Sci Biotechnol 15, 100 (2024)**

**https://doi.org/10.1186/s40104-024-01057-w**

Following publication of the original article [1], the authors reported errors in Fig. 2 and Table 2. In Fig. 2, CON group discrepancies in the arrangement and SIL1000 group in week4 used wrong figure that led to a misrepresentation of the visual data. Fig. 2 has been repositioned to align with the precise specifications outlined in the original article, and is corrected from:

Incorrect Fig. 2

**Fig. 2:**
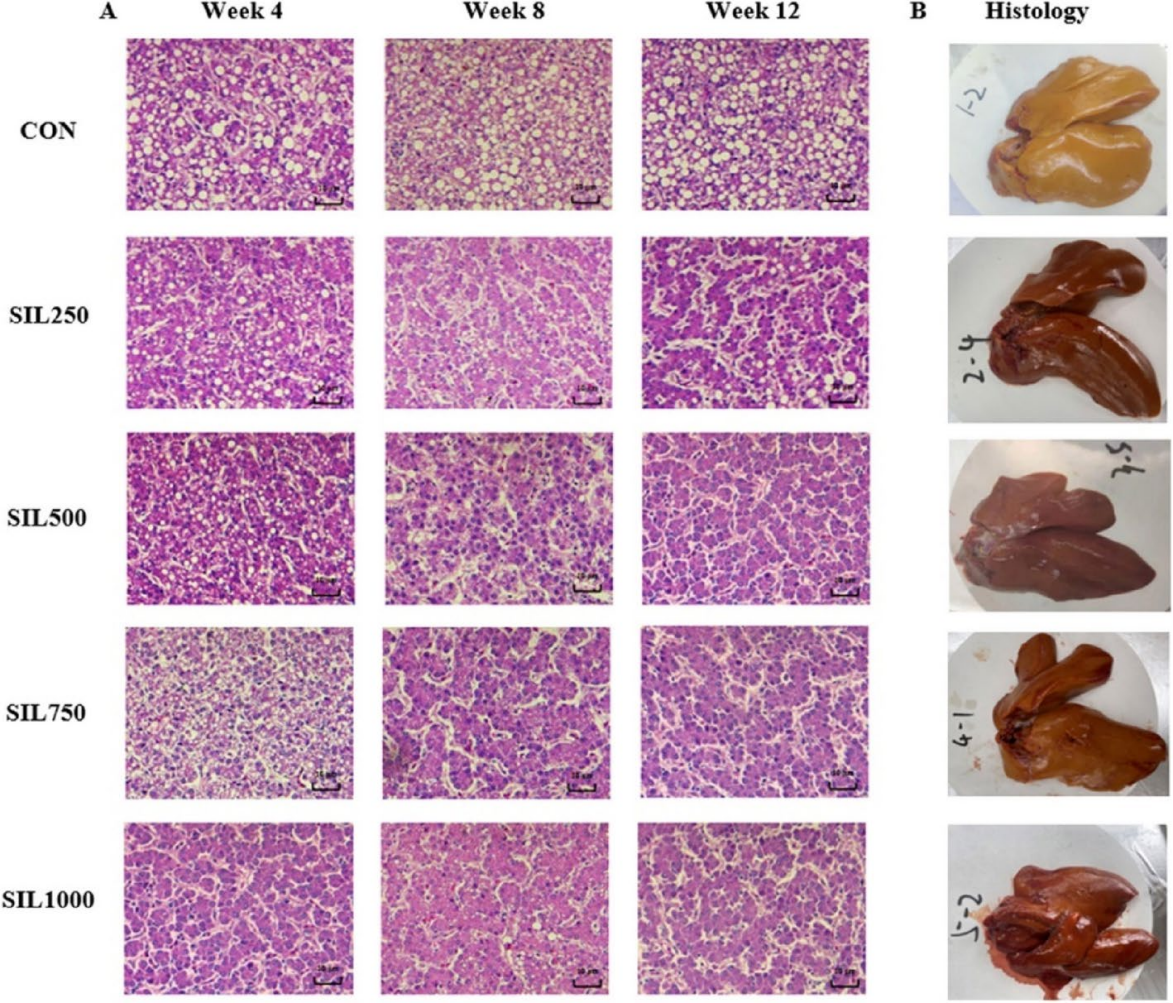
The effect of dietary silymarin on hepatic histomorphology of laying hens. **A** The effect of dietary silymarin on hepatic histomorphology H&E stained sections of laying hens (400×magnifcation). **B** Liver images at week 12

To correct Fig. 2

**Fig. 2:**
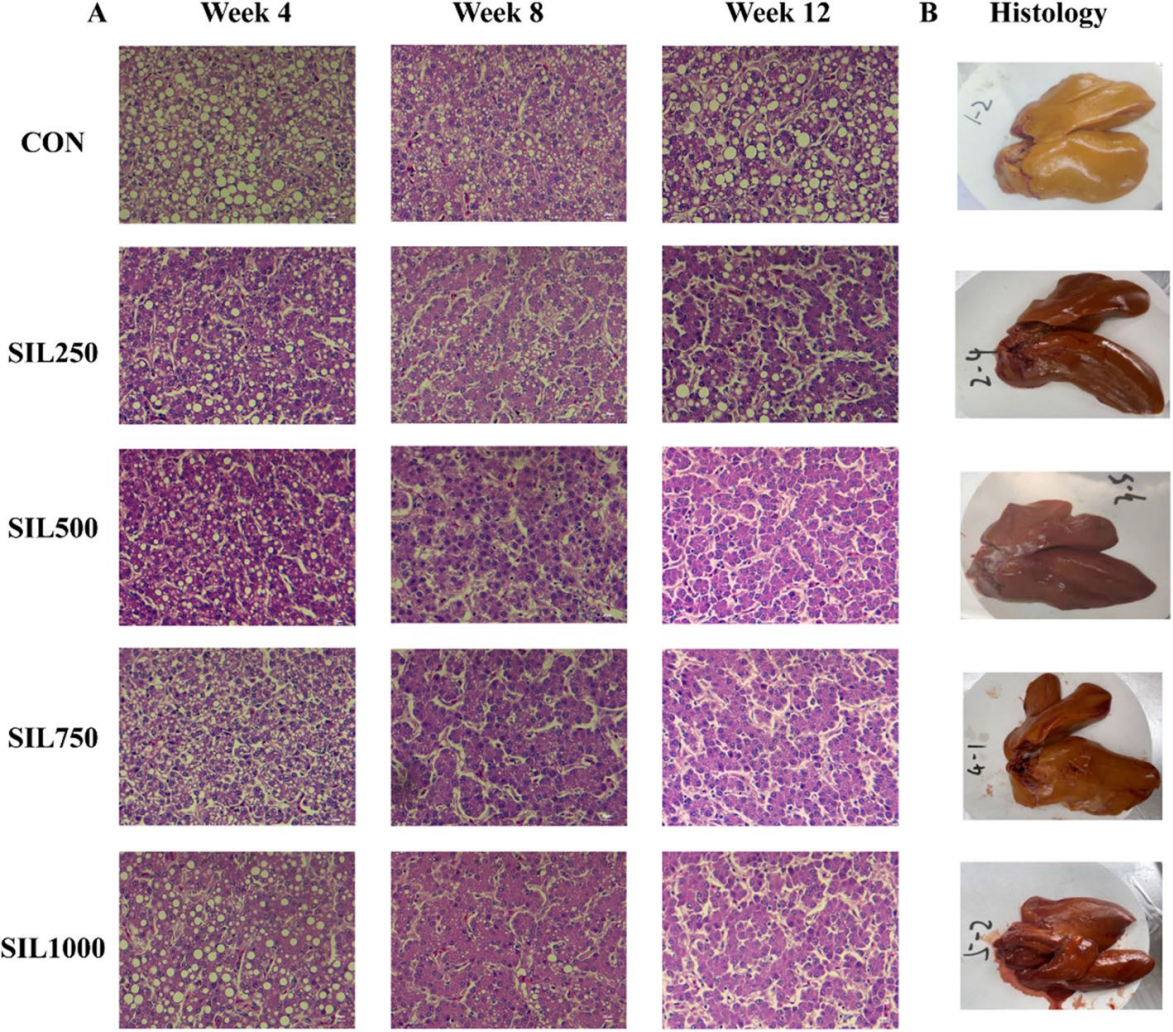
The effect of dietary silymarin on hepatic histomorphology of laying hens. **A** The effect of dietary silymarin on hepatic histomorphology H&E stained sections of laying hens (400×magnifcation). **B** Liver images at week 12

In Table 2, the incorrect BSEP gene information primers were used for RT-qPCR. Table 2 has been corrected from:

Incorrect Table 2

**Table 2:** Primers used for RT-qPCR

**Genes**	**Primer sequence (5´ → 3´)**	**GenBank ID**	**Product length, bp**
*β-actin*	Forward: CCAGCCATGTATGTAGCCATCC Reverse: CACCATCACCAGAGTCCATCAC	NM_205518.2	88
*FXR*	Forward: TGGAGGCAACTGTGAGATGG Reverse: TGCCCATTTGCTTGCATTTCC	NM_24113.3	80
*CYP7A1*	Forward: GATCTTCCCAGCCCTTGTGG Reverse: AGCCTCTCCCAGCTTCTCAC	NM_001001753.2	82
*BSEP*	Forward: TGGAATAGAGCGTGGCTTTT Reverse: CATTGGCAGTCATCTCAGGA	XM_046921957.1	120
*MRP2*	Forward: GGAGAGCAGTGATGCAAGTAGT Reverse: AGTTACTGAGCTGCCGATGC	NM_001012522.3	110
*ESR1*	Forward: TGGTACTACCGCTCCAGTGT Reverse: AGGCTGCTTGACCCAAAAGA	NM_205183.2	78
*ESR2*	Forward: TGCAGTGAACGACAAATTCAGA Reverse: TCCCTGTGAAGGCAAGACCT	NM_204794.3	82
*ACC*	Forward: ACATCCATCTTTGATGTGCT Reverse: AGGACATTCTGTTTGGGTG	XM_046929958.1	199
*FASN*	Forward: TGCTATGCTTGCCAACAGGA Reverse: ACTGTCCGTGACGAATTGCT	NM_205155.4	128
*SCD*	Forward: ACCTTAGGGCTCAATGCCAC Reverse: TCCCGTGGGTTGATGTTCTG	NM_204890.2	89
*SREBP-1*	Forward: TGGTGGTGGACGCCGAGAAG Reverse: GTCGTTGATGGATGAGCGGTAGC	NM_204126.3	134
*PPARα*	Forward: AGGCCAAGTTGAAAGCAGAA Reverse: TTTCCCTGCAAGGATGACTC	NM_001001464.1	155
*PPARγ*	Forward: TCTCCTGGCTTCTCTCAT Reverse: TGGGCTCCATAAAGTCAC	NM_001397666.1	116
*ELOVL6*	Forward: ACAAGGGCTTTTGGTGTCTCA Reverse: GGCCTACGGAGGCTTTTTGA	NM_001031539.2	101
*ELOVL7*	Forward: TTCACATGTGGTGCTCCATT Reverse: TGCTAAGGGCCATTTTCACCT	NM_001197310.1	177
*ApoB*	Forward: GGTTACTCCCACGATGGCAA Reverse: TCGCAGAAATGCCCTTCCTT	NM_001044633.2	120
*ApoVLDLII*	Forward: CTTAGCACCACTGTCCCTGAAGT Reverse: TGCATCAGGGATGACCAGC	NM_205483	81
*VTGII*	Forward: TTGCAAGCTGATGAACACACAC Reverse: GATTGCTTCATCTGCCAGGTC	NM_001031276	192
*GPR30*	Forward: AGGTCCAAGGATGTGCGCTGA Reverse: GTCGTAAGACCACGGCGGGA	NM_001162405.1	156

To correct Table 2

**Table 2:** Primers used for RT-qPCR

**Genes**	**Primer sequence (5´ → 3´)**	**GenBank ID**	**Product length, bp**
*β-actin*	Forward: CCAGCCATGTATGTAGCCATCC Reverse: CACCATCACCAGAGTCCATCAC	NM_205518.2	88
*FXR*	Forward: TGGAGGCAACTGTGAGATGG Reverse: TGCCCATTTGCTTGCATTTCC	NM_24113.3	80
*CYP7A1*	Forward: GATCTTCCCAGCCCTTGTGG Reverse: AGCCTCTCCCAGCTTCTCAC	NM_001001753.2	82
*BSEP*	Forward: TGCAAAGCAAAGGAGACT Reverse: GCAATGGATAATGGAGGG	XM_040676679.2	193
*MRP2*	Forward: GGAGAGCAGTGATGCAAGTAGT Reverse: AGTTACTGAGCTGCCGATGC	NM_001012522.3	110
*ESR1*	Forward: TGGTACTACCGCTCCAGTGT Reverse: AGGCTGCTTGACCCAAAAGA	NM_205183.2	78
*ESR2*	Forward: TGCAGTGAACGACAAATTCAGA Reverse: TCCCTGTGAAGGCAAGACCT	NM_204794.3	82
*ACC*	Forward: ACATCCATCTTTGATGTGCT Reverse: AGGACATTCTGTTTGGGTG	XM_046929958.1	199
*FASN*	Forward: TGCTATGCTTGCCAACAGGA Reverse: ACTGTCCGTGACGAATTGCT	NM_205155.4	128
*SCD*	Forward: ACCTTAGGGCTCAATGCCAC Reverse: TCCCGTGGGTTGATGTTCTG	NM_204890.2	89
*SREBP-1*	Forward: TGGTGGTGGACGCCGAGAAG Reverse: GTCGTTGATGGATGAGCGGTAGC	NM_204126.3	134
*PPARα*	Forward: AGGCCAAGTTGAAAGCAGAA Reverse: TTTCCCTGCAAGGATGACTC	NM_001001464.1	155
*PPARγ*	Forward: TCTCCTGGCTTCTCTCAT Reverse: TGGGCTCCATAAAGTCAC	NM_001397666.1	116
*ELOVL6*	Forward: ACAAGGGCTTTTGGTGTCTCA Reverse: GGCCTACGGAGGCTTTTTGA	NM_001031539.2	101
*ELOVL7*	Forward: TTCACATGTGGTGCTCCATT Reverse: TGCTAAGGGCCATTTTCACCT	NM_001197310.1	177
*ApoB*	Forward: GGTTACTCCCACGATGGCAA Reverse: TCGCAGAAATGCCCTTCCTT	NM_001044633.2	120
*ApoVLDLII*	Forward: CTTAGCACCACTGTCCCTGAAGT Reverse: TGCATCAGGGATGACCAGC	NM_205483	81
*VTGII*	Forward: TTGCAAGCTGATGAACACACAC Reverse: GATTGCTTCATCTGCCAGGTC	NM_001031276	192
*GPR30*	Forward: AGGTCCAAGGATGTGCGCTGA Reverse: GTCGTAAGACCACGGCGGGA	NM_001162405.1	156

This correction does not alter the overall conclusions or findings of the study.

The original article [1] has been updated.
