# Supplementary material for: Dietary silymarin improves performance by altering hepatic lipid metabolism and cecal microbiota function and its metabolites in late laying hens
Source: J Anim Sci Biotechnol. 2024 Jul 13;15:100. doi: 10.1186/s40104-024-01057-w (PMC11245868; doi:10.1186/s40104-024-01057-w)
Supplement: Supplementary file 1 — Additional file 1: Fig. S1. Ingredient testing of SIL. Table S1. Average laying rate (pre-feeding period). [file 40104_2024_1057_MOESM1_ESM.docx]

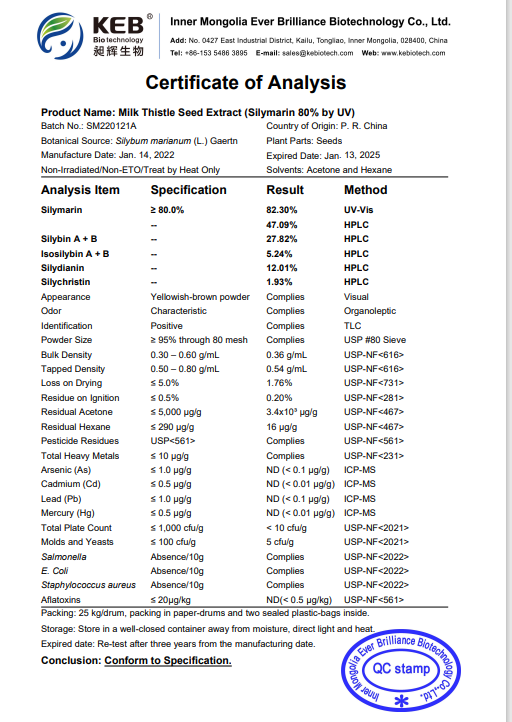


**Fig. S1** Ingredient testing of SIL

**Table S1** Average laying rate (pre-feeding period)

| **Group** | **Average laying rate , % (pre-feeding period)** |
| --- | --- |
| CON | 93.32 |
| CON | 95.26 |
| CON | 91.02 |
| CON | 91.21 |
| CON | 92.12 |
| CON | 91.22 |
| SIL250 | 89.23 |
| SIL250 | 94.21 |
| SIL250 | 92.82 |
| SIL250 | 89.01 |
| SIL250 | 90.11 |
| SIL250 | 89.92 |
| SIL500 | 93.22 |
| SIL500 | 94.42 |
| SIL500 | 90.72 |
| SIL500 | 92.21 |
| SIL500 | 94.25 |
| SIL500 | 93.21 |
| SIL750 | 92.33 |
| SIL750 | 92.52 |
| SIL750 | 95.44 |
| SIL750 | 92.14 |
| SIL750 | 93.22 |
| SIL750 | 92.67 |
| SIL1000 | 89.36 |
| SIL1000 | 94.68 |
| SIL1000 | 93.22 |
| SIL1000 | 88.89 |
| SIL1000 | 92.21 |
| SIL1000 | 95.21 |
